# Bibliometric Mapping of Quercetin Research: Analysis of the Most‐Cited Articles (2000–2023)

**DOI:** 10.1002/fsn3.70500

**Published:** 2025-07-10

**Authors:** Aijuan Yang, Jiyun Wang, Wei Yang, Zhengfei Yang, Liming Zhang, Hangying Li

**Affiliations:** ^1^ People's Hospital of Ningxia Hui Autonomous Region Ningxia Medical University Yinchuan P. R. China; ^2^ School of Pharmacy Ningxia Medical University Yinchuan P. R. China; ^3^ School of Traditional Chinese Medicine Ningxia Medical University Yinchuan P. R. China

**Keywords:** bibliometric analysis, most‐cited articles, quercetin, VOSviewer, web of science

## Abstract

Quercetin, a naturally occurring flavonoid with demonstrated antioxidant, anti‐inflammatory, and anticancer properties, has emerged as a focal point in biomedical research. Despite extensive studies, a systematic bibliometric analysis of quercetin research remains lacking. To visualize research trends, collaboration networks, and knowledge domains of quercetin research, we analyzed the top 200 most‐cited articles from the Web of Science Core Collection (2000–2023) using VOSviewer software to visualize research trends, collaboration networks, and knowledge domains. The analysis identified China (*n* = 44; 11,238 citations) and the United States (*n* = 33; 8254 citations) as the leading contributors, with Tokushima University emerging as the most productive institution (*n* = 6; 1800 citations). The most influential research team was the Wolffram, Siegfried, and Hollman, PCH group. The Journal of Agricultural and Food Chemistry emerged as the top publishing journal (*n* = 14; 3738 citations). Science mapping through keyword co‐occurrence analysis identified four primary research hotspots: (1) molecular mechanisms and delivery systems, (2) therapeutic applications, (3) active constituents and analytical methods, and (4) metabolic and cellular regulation. Major research topics included cancer (*n* = 31; 7271 citations), antioxidant effects (*n* = 20; 6513 citations), and neurological diseases (*n* = 17; 3544 citations). The dominant research areas were biochemistry and molecular biology (*n* = 40; 9997 citations) and pharmacology and pharmacy (*n* = 39; 11,208 citations). The study maps the field's intellectual structure, collaboration dynamics, and developmental trajectories, providing clear identification of knowledge gaps and strategic direction for future research in this rapidly evolving field.

## Graphical abstract

This study performs a bibliometric analysis of the 200 most‐cited articles on quercetin, identifying key research hotspots, topics, leading institutions, and countries, while emphasizing the global and interdisciplinary nature of quercetin research. It also reveals current research trends and challenges in the field, providing a thorough overview of its complexities. Finally, the analysis offers insights into research trends and gaps, guiding the direction of future studies in this crucial area.

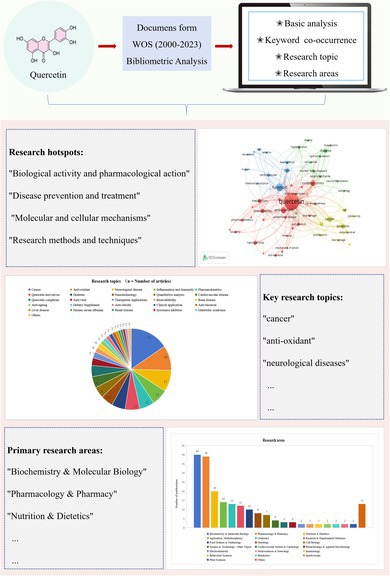

**Abbreviations**
HPLC: High‐Performance Liquid Chromatography
HPLC‐MS: High‐Performance Liquid Chromatography‐Mass Spectrometry
JAK/STAT: Janus kinase‐signal transducer and activator of transcription
MAPK: Mitogen‐Activated Protein Kinases
MAPK/Nrf2: Mitogen‐Activated Protein Kinases/Nuclear factor erythroid 2‐related factor 2
MEK/ERK: Mitogen‐Activated Protein Kinases/Extracellular signal‐regulated kinases
NF‐κB: Nuclear factor kappa‐B
Nrf2/Keap1: Nuclear factor erythroid 2‐related factor/Kelch‐like ECH‐associated protein 1
PI3K/AKT/mTOR: Phosphatidylinositol‐3 kinase/Protein Kinase B/Mammalian Target Of Rapamycin
Wnt/β‐catenin: Canonical Wnt/β‐catenin pathway

## 1 Introduction

Natural compounds have long served as a crucial reservoir of bioactive molecules associated with human health and disease prevention. Within this context, flavonoids, a substantial class of polyphenolic compounds, have received extensive academic attention for their remarkable antioxidant properties and potential therapeutic applications. Notably, quercetin, which is ubiquitous in nature and possesses a wide range of pharmacological activities and health benefits, has become one of the most widely studied flavonoids. Quercetin is particularly abundant in a variety of fruits and vegetables, including capers (with the highest concentration of 234 mg/100 g), onions, apples, and St. John's wort ( *Hypericum perforatum* ), as well as berries, mangoes, asparagus, Brassica vegetables (such as kale and cabbage), tea leaves, nuts, and seeds. It is also derived from numerous medicinal plants including *Ginkgo biloba* , *Mentha canadensis* , and *Crataegus pinnatifida* (Ozorowski et al. 2025; Baiomy 2024). It possesses a wide range of biological activities, such as antioxidant (Kılıç et al. 2025), anti‐inflammatory (Hou et al. 2019), antibacterial (Shastri et al. 2025), antiviral (Mukherjee and Tripathi 2025), antitumor (Wang et al. 2024), anti‐aging (Abbiati et al. 2023), and immunomodulatory effects (Michalski et al. 2020), as well as hepatoprotective (Atta et al. 2025) and cytotoxic activities (Milanezi et al. 2019). Quercetin mediates these activities by modulating various cellular signaling pathways, such as NF‐κB (Seker et al. 2024; Banerjee et al. 2022), MAPK (Min et al. 2019), PI3K/AKT (Liu et al. 2024), JAK/STAT (Zalpoor et al. 2022), Wnt/β‐catenin (Mi et al. 2022), and so forth. Through these mechanisms, it plays a critical role in the prevention and treatment of diseases, including cardiovascular and cerebrovascular disorders (Dabeek and Marra 2019), neurodegenerative diseases (Khan et al. 2019), tumors, and cancers (Rauf et al. 2018). Despite its well‐established bioactive properties, quercetin's clinical utility remains constrained by significant pharmacological limitations. Its poor aqueous solubility and low bioavailability severely restrict therapeutic efficacy (Ai et al. 2024). Moreover, these challenges are compounded by the compound's instability during food processing and storage, with marked susceptibility to degradation from environmental factors including light, heat, and pH variations (Albuquerque et al. 2021; Ren et al. 2020). Additionally, quercetin undergoes extensive first‐pass metabolism in the gastrointestinal tract and liver, substantially reducing its systemic bioavailability and target tissue accumulation (Ulusoy and Sanlier 2020). Collectively, these physicochemical and pharmacokinetic barriers have significantly hindered both its development as a therapeutic agent and its effective incorporation into functional food formulations (Zhao et al. 2025). To overcome these challenges, researchers have conducted systematic investigations into the synthesis of quercetin and its derivatives, alongside developed diverse drug delivery systems aimed at enhancing its bioavailability and therapeutic efficacy (Ghanbari‐Movahed et al. 2022). The extensive application of nanotechnology in quercetin research has also provided innovative solutions to improve its delivery efficiency and bioactivity (Eity et al. 2024). Over the past two decades (2000–2023), quercetin has become a central subject in a substantial body of research literature, as evidenced by Figure S1 showing its remarkable growth trajectory from just 50 annual publications in 2000 to over 600 in 2023, representing a 12‐fold increase, and Figure S2 demonstrating its multidisciplinary research landscape spanning Pharmacology and Pharmacy, Biochemistry and Molecular Biology, and Food Science and Technology. (Figures S1 and S2 source: WoSCC database; Figures S1 and S2). Academic databases presently contain tens of thousands of quercetin‐related articles, with the volume of such publications rising every year. This trend underscores the growing interest in quercetin within the research community. The exponential increase in the volume of publications has rendered it difficult to track the progression of the field and discern which studies have had a significant impact. Despite over 500 published review articles synthesizing various aspects of quercetin research (Boots et al. 2008; Vollmannová et al. 2024), the field still lacks a systematic mapping of the knowledge domain through bibliometric analysis, particularly regarding the evolutionary trajectory and intellectual structure revealed by most‐cited articles (2000–2023). This study fills this critical gap by using bibliometric and science mapping analysis to visualize research trends, identify knowledge clusters, and analyze the most influential publications that have shaped quercetin research over the past two decades.

Bibliometric analysis is extensively used in medical and healthcare research (Ninkov et al. 2022; Hassan and Duarte 2024), as it assesses publication quantity, quality, citation trends, and citation relationships of publications by analyzing the scientific literature. This approach provides quantitative and qualitative insights, facilitating a comprehensive understanding of the structure, impact, and dynamics of academic communication. Moreover, it is particularly adept at uncovering trends within specific fields and identifying emerging research hotspots. Additionally, such analysis can evaluate the influence of researchers and institutions as well as the impact of their scholarship. Over time, bibliometric analysis has now evolved into a scientific discipline that plays an integral role in research methodology and contributes significantly to the assessment and comprehension of the scope and significance of academic work. Specifically, this analytical method can generate valuable graphical representations that identify the most research‐productive regions, countries, and institutions, reveal the geographical distribution of the most rigorously designed studies, and demonstrate collaborative networks among researchers, institutions, and across borders (Watson and Hayter 2025). Citation analysis serves as a fundamental method within the field of bibliometrics (Ellegaard and Wallin 2015). The citation count of an academic article is a significant criterion for measuring its influence and academic value (Moed 2009). The most extensively cited studies are also core papers of superior methodological quality, hence of notable scientific merit (Panayi et al. 2023). However, citation frequency alone does not necessarily reflect the quality of an article. There are different views on what constitutes a “classic article” (Ahmad et al. 2019; Karabay et al. 2024). Some scholars regard articles with over 400 citations as classic literature whereas in certain disciplines, particularly within the medical field, articles with over 100 citations may also be considered classics. In general, articles that receive a substantial number of citations are esteemed within the scientific community, as they introduce novel concepts or address critical issues (He et al. 2021). A substantial number of citations indicates that an article has had a significant impact on the advancement of knowledge.

Bibliometric analysis has been extensively utilized in the field of food and medicine, including analyzing research on herbal medicines and individual compound studies for cancer treatment (Bai et al. 2023; Fu et al. 2024), examining global research trends related to specific natural products (Dong et al. 2022), and systematically analyzing and categorizing highly cited articles related to particular compounds (Zhou et al. 2022). A notable example of this application is the study by Zhou YX and colleagues, who analyzed the 100 most‐cited articles on curcumin. Their analysis elucidated the principal features and research hotspots within these articles, offering the academic community a comprehensive view of the state of curcumin research. This study employs bibliometric and science mapping analysis to systematically evaluate the 200 most‐cited articles on quercetin research. By examining publication trends, collaborative networks, and keyword evolution, we aim to (1) map the knowledge structure of the field, (2) highlight key contributions from leading institutions, countries, and journals, (3) identify emerging research hotspots and topics, and (4) elucidate critical opportunities and challenges for future quercetin research. These findings will provide a data‐driven foundation to understand the evolving landscape of quercetin studies while offering valuable insights to guide subsequent investigations in this field.

## 2 Materials and Methods

### 2.1 Search Strategy

A systematic literature review was conducted using the Science Citation Index Expanded (SCI‐E) within the Web of Science Core Collection (WoSCC) database on September 24, 2024. To enhance the precision of the search results (Yang et al. 2022), the search strategy employed the term “Quercetin” within the Title, Abstract, and Author Keywords fields. The literature search encompassed publications from January 1, 2000, to December 31, 2023. The inclusion criteria were strictly limited to English‐language original research articles and reviews, while systematically excluding: retracted publications, proceeding papers, editorial material, letters, book chapters, publications with expression of concern, early access items, duplicate studies, and studies unrelated to quercetin. All eligible records were ranked by citation frequency, and the top 200 most‐cited articles were selected for bibliometric analysis after independent validation by two authors to confirm thematic relevance to quercetin research and citation accuracy.

### 2.2 Data Extraction and Analysis

For the analysis and visualization of articles concerning quercetin, VOSviewer version 1.6.20 (https://www.vosviewer.com) and Microsoft Excel 2019 were utilized. In the keyword co‐occurrence network analysis, the minimum cluster size parameter was set to 9 for author keywords and 10 for Keywords Plus. For the country/author collaboration network analysis, the minimum cluster size was set to 1. All other parameters maintained the default settings of VOSviewer software (1.6.20). The following information was extracted and analyzed for the included papers: annual number of publications, per year total citations, citation frequency, country, institutions, author, journal, keywords (author keywords and Keywords Plus), research topic, and research areas.

VOSviewer is a robust open‐source software application for bibliometric analysis (Bukar et al. 2023). It processes large‐scale datasets and visualizes them, supporting various data formats. VOSviewer provides extensive network data mapping and visualization tools, including clustering analysis, keyword co‐occurrence network analysis, and density views. These features enable researchers to conduct in‐depth analyses and gain insights into development trends and research hotspots within the scientific literature (van Eck and Waltman 2010). While VOSviewer offers unique advantages for bibliometric analysis, studies by van Eck and Waltman (2010) and Bukar et al. (2023) have identified several key limitations: (1) Visualization is limited to two‐dimensional distance mapping based on VOS technology, lacking multi‐dimensional display and complex graph structure analysis capabilities; (2) Limited text processing features, particularly the absence of stemming and temporal analysis functions; (3) Stringent data compatibility requirements necessitating specific formatting for unstructured data preprocessing; and (4) Primary design for traditional bibliometric scenarios, with potential performance constraints when handling emerging data types like social media content. These findings suggest that researchers should select complementary analytical tools based on specific research needs.

As outlined in the “VOSviewer Manual (1.6.20),” the size of the nodes (circles) in a network map generated by VOSviewer generally indicates the number of publications associated with entities such as countries, institutions, authors, or journals, or reflects the frequency of keyword occurrences. The lines (edges) represent the relationships connecting nodes, with their thickness typically denoting the strength of these relationships, such as the frequency of co‐occurrence. Colors distinguish between clusters, where nodes sharing the same color generally belong to the same cluster, indicating a degree of relatedness or similarity.

## 3 Results

### 3.1 Basic Characteristics

The search strategy yielded a total of 6099 papers from the WoSCC. After applying filters for publication language and document type, 6021 articles were identified. These articles were ranked in descending order of citation frequency, resulting in the selection of the top 200 documents with the highest citation rates. Among these, 158 were research articles and 42 were review articles. The titles, first authors, document types, publishing journals, 5‐year journal impact factors, publication years, total citations, and annual average citations of the top 200 articles are summarized in Table S1.

### 3.2 Publication Year Analysis

**FIGURE 1:**
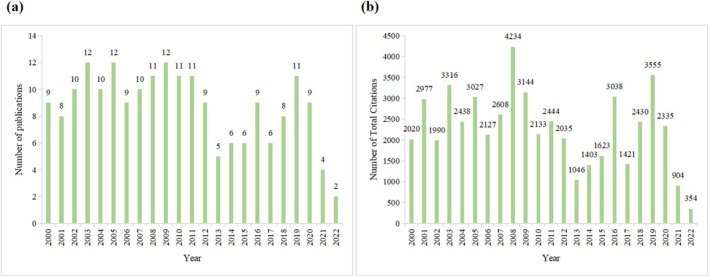
Annual number of publications (a) and citations (b) of the 200 most‐cited articles on quercetin.

The 200 most‐cited articles were published between 2000 and 2022. Notably, no articles published in 2023 achieved high citation counts likely due to insufficient time for recent publications to accumulate citations. Figure 1a illustrates the distribution of these articles over a 23‐year period. The horizontal axis represents the years, while the vertical axis indicates the annual number of published articles. The peak publication years were 2003, 2005, and 2009, each with 12 articles. This was followed by 11 articles published in 2008, 2010, 2011, and 2019. The lowest publication count occurred in 2022, with only 2 articles, likely reflecting the limited time for recent works to gain significant citations. In summary, the data reveal a fluctuating trend in the number of highly cited articles on quercetin research over the past 23 years, providing a visual depiction of academic engagement in this field.

### 3.3 Citation Analysis

Among the 200 most‐cited articles, citation counts ranged from 154 to 1409, with a median of 208 and an average of 263. Forty‐seven articles had over 300 citations, 14 exceeded 500, and 2 surpassed 1000. The highest annual citation count was recorded in 2008, with 4234 citations, coinciding with the publication of the most‐cited article. This was followed by 2019 (3555 citations), 2003 (3316 citations), and 2009 (3114 citations). Figure 1b presents the annual cumulative citation counts for the 200 most‐cited articles on quercetin.

Our analysis identified the work by Boots, A.W., et al., titled “Health effects of quercetin: from antioxidant to nutraceutical,” published in the *European Journal of Pharmacology* in 2008, as the most‐cited article, with 1409 citations. This article explores the potential of quercetin in preventing osteoporosis, cancer, and cardiovascular diseases as well as its anti‐aging properties (Boots et al. 2008). It provides a comprehensive review of the health benefits of quercetin, elucidating its antioxidant mechanisms and its applications as a nutraceutical.

Further analysis of the top 10 most‐cited articles among the 200 highly cited publications revealed three primary research dimensions: (1) fundamental mechanisms of quercetin (e.g., antioxidant and anti‐inflammatory properties); (2) methodological innovations (e.g., clinical combination therapies); and (3) translational applications (e.g., multi‐target drug development). However, in‐depth evaluation identified notable limitations: First, 60% were review articles focusing on theoretical synthesis rather than original discoveries; second, only one clinical research paper was included, indicating a significant gap in bench‐to‐bedside translation; finally, the absence of recent studies in this top‐cited cohort may not adequately reflect current research frontiers. These findings suggest the need for cautious interpretation regarding the representativeness of highly cited literature.

### 3.4 Country Analysis

**FIGURE 2:**
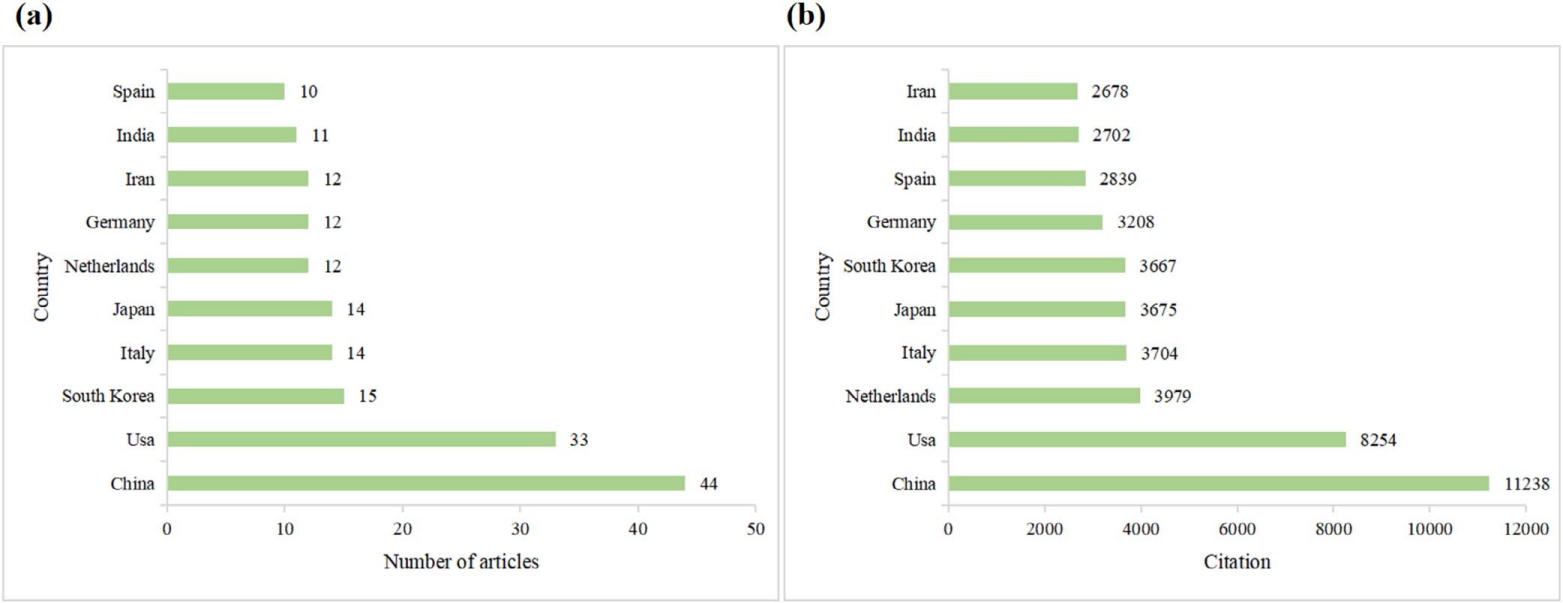
The number of articles (a) and citation counts (b) of the top 10 countries.

A total of 47 countries or regions contributed to the 200 most‐cited articles. It is important to note that a high volume of publications does not inherently correlate with a high citation count; hence, separate analyses were conducted for publication output and citation counts. The analysis focused on the top 10 countries, ranked by both publication output and citation counts. Table 1 ranks these countries based on the number of published articles and their citation counts. China, the United States, and South Korea were the leading nations in publication output, with 44, 33, and 15 articles, respectively. By citation counts, China, the United States, and the Netherlands occupied the top three positions, with 11,238, 8254, and 3979 citations, respectively. Figure 2a,b provide a comparative analysis of the number of articles and citation counts for these top 10 countries.

**TABLE 1:** The number of articles and citation counts of the top 10 countries.

No.	Number of articles	Country	Citation	Country
1	44	China	11,238	China
2	33	Usa	8254	Usa
3	15	South Korea	3979	Netherlands
4	14	Italy	3704	Italy
5	14	Japan	3675	Japan
6	12	Netherlands	3667	South Korea
7	12	Germany	3208	Germany
8	12	Iran	2839	Spain
9	11	India	2702	India
10	10	Spain	2678	Iran

**FIGURE 3:**
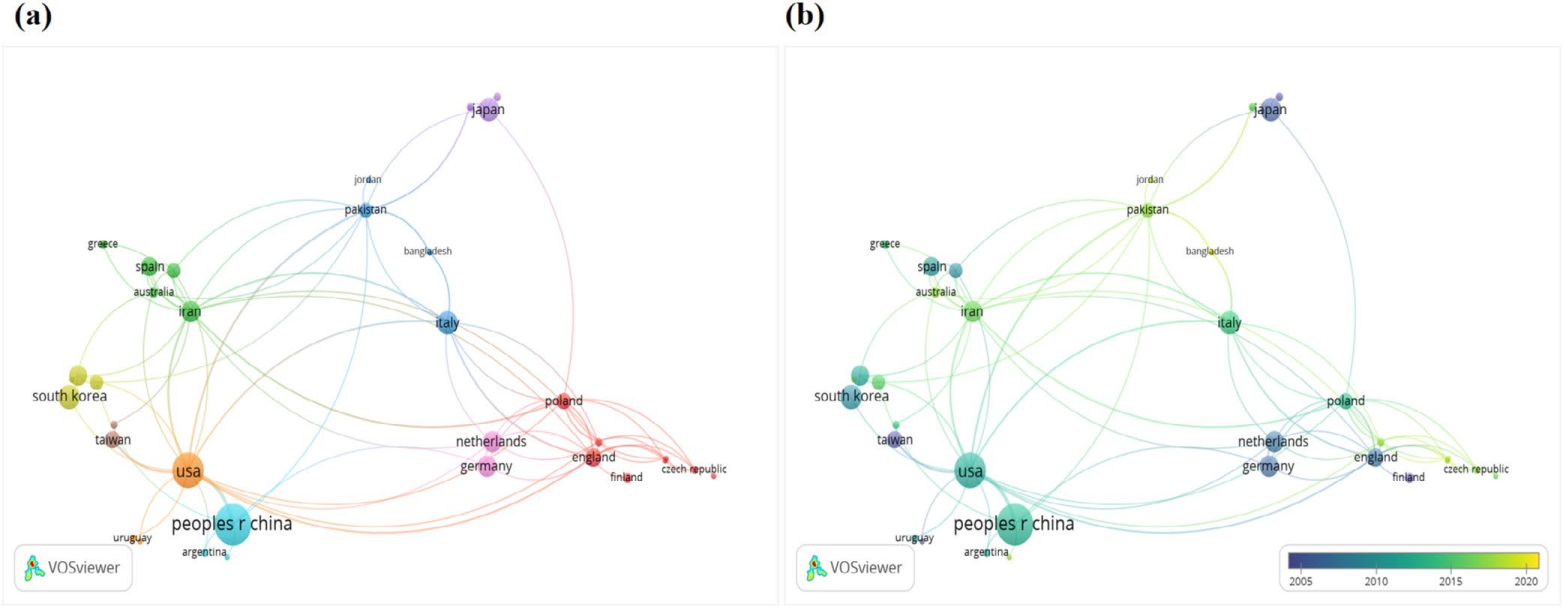
Map of co‐authorship between countries. (a) Network visualization map showing cluster analysis of countries associated with quercetin and (b) overlay visualization map showing trends of country frequency over time.

The VOSviewer analysis of international collaboration highlights robust cooperative networks among the United States, the United Kingdom, Pakistan, Poland, and China. The network of collaborations among these countries is depicted in Figure 3a,b. As evidenced by the highest node centrality scores (Figure 3a), China and the United States serve as pivotal hubs in quercetin research, aligning with their dominant scientific output shown in Figure 2. The overlay visualization (Figure 3b) further reveals a marked increase in transnational collaboration density post‐2015, suggesting the emergence of a globalized research phase for quercetin. The co‐authorship network analysis among countries reveals relatively limited international collaborations at present, highlighting the need for enhanced global research cooperation in future studies.

### 3.5 Institutions Analysis

VOSviewer analysis identified 339 institutions that contributed to the 200 most‐cited articles on quercetin. Table 2 presents the publication quantities and citation counts of these institutions. When ranked by both the number of published articles and citation counts, Tokushima University in Japan (*n* = 6; 1800 citations), the University of Kiel in Germany (*n* = 6; 1643 citations), and Shandong University in China (*n* = 6; 1508 citations) consistently occupy the top three positions. These institutions have made significant contributions to quercetin research.

**TABLE 2:** The number of articles and citation counts of the top 10 institutions.

No.	Number of articles	Institution (Country)	Citation	Institution (Country)
1	6	Tokushima University (Japan)	1800	Tokushima University (Japan)
2	6	University of Kiel (Germany)	1643	University of Kiel (Germany)
3	6	Shandong University (China)	1508	Shandong University (China)
4	5	Complutense University of Madrid (Spain)	1436	Chinese Academy of Sciences (China)
5	5	Baqiyatallah University of Medical Sciences (Iran)	1409	Maastricht University (Netherlands)
6	4	Nanjing University (China)	1319	Northeast Forestry University (China)
7	3	Chinese Academy of Sciences (China)	1255	Complutense University of Madrid (Spain)
8	3	Universidad de Leon (Spain)	1010	Baqiyatallah University of Medical Sciences (Iran)
9	3	Wageningen University & Research (Netherlands)	1008	Universidad de Leon (Spain)
10	3	Consiglio Nazionale delle Ricerche (Italy)	978	Wageningen University & Research (Netherlands)

### 3.6 Author Analysis

The volume of scientific publications by individual authors serves as an indicator of their contributions to their respective fields (Wu et al. 2021). However, an author's impact should also be evaluated based on the citation counts of their work, not just the quantity. To address this dual need, the *H*‐index—a composite metric in scientometrics—was proposed in 2005 by American physicist Jorge E. Hirsch (Hirsch 2005). It integrates two key dimensions (Kamrani et al. 2021): a researcher's publication count (reflecting productivity) and the citation count of those publications (reflecting scholarly influence).

**FIGURE 4:**
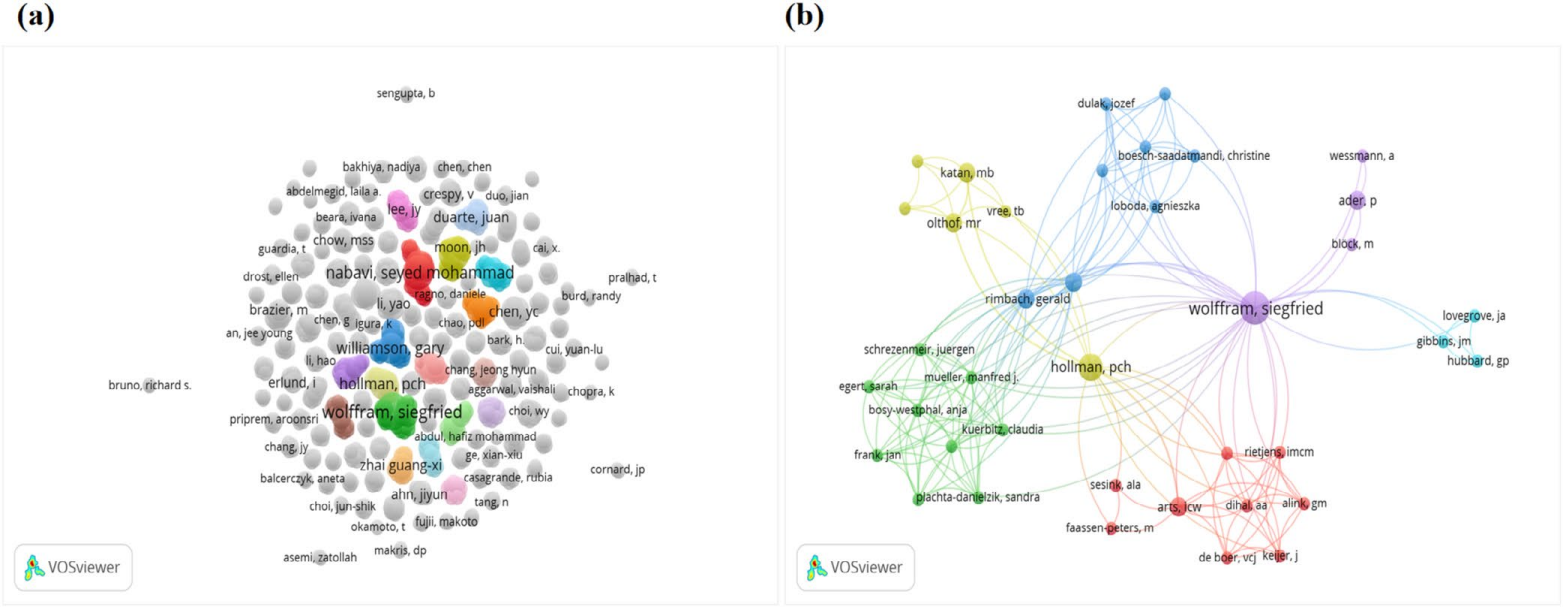
Network visualization map showing cluster analysis of authors. (a) Does not show the direct connections between 1030 authors. (b) Shows the linking relationships among 39 authors.

For the 200 most‐cited articles on quercetin, 1030 authors were involved. Among these, 15 authors published more than three articles, with four of these authors based in the Netherlands. Table S2 lists the authors who published three or more articles. The author with the highest output was Wolffram Siegfried from the University of Kiel, with six articles. The next most prolific authors, each with five articles, were Nabavi Seyed Mohammad from Baqiyatallah University of Medical Sciences, Terao Junji from Tokushima University, and Williamson Gary from the University of Leeds. When ranked by citation counts, Bast Aalt, Boots Agnes W., and Haenen Guido R. M. M. from Maastricht University lead with 1777 citations. Wolffram Siegfried ranks second with 1643 citations. According to *H*‐index rankings, the three highest‐ranking authors are Wolffram Siegfried, Terao Junji, and Hollman PCH, with *H*‐indices of 31, 31, and 13, respectively. Table 3 provides details of the top 10 authors, including publication numbers, citation counts, and *H*‐indices. VOSviewer analysis of author collaboration revealed a network centered on four principal authors: Wolffram Siegfried, Williamson Gary, Nabavi Seyed Mohammad, and Hollman PCH, among which Wolffram Siegfried and Hollman PCH exhibited the highest node degrees, indicating their dominant roles as hubs in quercetin research collaboration. Figure 4a,b illustrate this network, highlighting clusters of collaborative relationships. Based on a comprehensive evaluation of publication numbers, citation counts, *H*‐index, and co‐authorship networks, we have identified Wolffram Siegfried and Hollman PCH as the two most influential contributors to quercetin research.

**TABLE 3:** The number of articles and citation counts of the top 10 authors.

No.	Number of articles	Author (Country)	*H*‐index	Citation	Author (Country)	*H*‐index
1	6	Wolffram, Siegfried (Germany)	31	1777	Bast, Aalt (Netherlands)	12
2	5	Nabavi, Seyed Mohammad (Iran)	9	1777	Haenen, Guido R. M. M. (Netherlands)	11
3	5	Terao, Junji (Japan)	31	1777	Boots, Agnes W. (Netherlands)	9
4	5	Williamson, Gary (England)	12	1643	Wolffram, Siegfried (Germany)	31
5	4	Hollman, PCH (Netherlands)	13	1319	Li, Yao (China)	8
6	4	Nabavi, Seyed Fazel (Iran)	8	1319	Yang, Jiaxin (China)	5
7	3	Bast, Aalt (Netherlands)	12	1319	Wang, Shengnan (China)	4
8	3	Gonzalez‐Gallego, Javier (Spain)	12	1319	Yao, Jiaying (China)	3
9	3	Haenen, Guido R. M. M. (Netherlands)	11	1319	Chaudhry, Maria Tabassum (China)	2
10	3	Boots, Agnes W. (Netherlands)	9	1192	Hollman, PCH (Netherlands)	13

### 3.7 Journal Analysis

The 200 most‐cited articles on quercetin were published in 115 academic journals. The *Journal of Agricultural and Food Chemistry* had the highest number of publications (14), followed by *Free Radical Biology* and *Medicine* and the *Journal of Nutrition*, each with seven articles. The *Journal of Agricultural and Food Chemistry* also garnered the highest citation count (3738), followed by the *European Journal of Pharmacology* (2388) and *Nutrients* (1894).

The journal impact factor (JIF) serves as a pivotal metric for evaluating academic journal influence. It is calculated by dividing the total citations received in the current year to articles published in the previous 2 years by the number of citable items published during that 2‐year period. The 5‐Year impact factor extends this calculation to a 5‐year window. Generally, higher impact factors indicate greater scholarly influence within a field. The impact factors (IF) of these journals ranged from 1.5 to 28, with a median value of 4.6. Among the 200 articles, 12 appeared in journals with an IF above 10 and 38 in journals with an IF above 5. The impact factor for each journal is detailed in Table S1. Based on the journal citation reports (JCR), the 115 journals were distributed across quartiles: 58 in Q1, 35 in Q2, 19 in Q3, and 3 in Q4. Table 4 details the number of articles and citations for the top 10 journals, while Table S3 provides additional information about these journals. As evidenced in Table S3, the major journals in quercetin research predominantly fall within JCR Q1/Q2 categories. Notably, 11 out of the 12 listed journals maintain impact factors exceeding 4.0, demonstrating their established authority in disseminating significant findings in this research domain. This significant correlation between journal impact factors (Table S3) and the dual metrics of publication volume and citation counts among leading journals (Table 4) substantiates their pivotal contribution to quercetin research advancement.

**TABLE 4:** The number of articles and citation counts of the top 10 journals.

No.	Number of articles	Journal	Citation	Journal
1	14	Journal of agricultural and food chemistry	3738	Journal of agricultural and food chemistry
2	7	Free radical biology and medicine	2388	European journal of pharmacology
3	7	Journal of nutrition	1894	Nutrients
4	6	Biochemical pharmacology	1854	Journal of nutrition
5	6	Life sciences	1778	Free radical biology and medicine
6	5	European journal of pharmacology	1636	Biochemical pharmacology
7	5	Phytotherapy research	1330	Molecules
8	4	Food chemistry	1236	Life sciences
9	4	Journal of nutritional biochemistry	1146	Phytotherapy research
10	4	Molecules	929	British journal of nutrition

### 3.8 Keyword Co‐Occurrence Analysis

Author keywords, as designated by the original authors, comprise a set of terms that the authors consider to most accurately encapsulate the content of their manuscript (Zhang et al. 2016; Lu et al. 2020). These keywords serve to articulate the primary themes addressed in their full‐text articles. Analyzing the most frequently employed author keywords is an essential methodological approach for identifying emerging trends and focal areas of scholarly inquiry within a specific discipline (Tripathi et al. 2018; Zhan et al. 2024).

Keywords Plus generated by an algorithm developed by Clarivate analytics are terms or phrases that frequently occur in the titles of an article's references as identified by an automated computer algorithm (Tripathi et al. 2018). These terms may not appear in the article's title or be listed as author keywords yet they are intended to be more descriptive than traditional keywords thereby providing a more concise representation of the paper's content (Nagendrababu et al. 2022; Garfield and Sher 1993). Keywords plus delivers a wide‐ranging view and proves useful for analyses at the field level (Gupta and Singh 2025).

As both Keywords Plus and Concepts are valuable tools in bibliometric analysis, each possesses distinct advantages (Gupta and Singh 2025). Considering the unique characteristics of author keywords and Keywords Plus, distinct analyses were performed for each to identify research hotspots in quercetin studies using VOSviewer. This methodological approach enabled us to investigate and compare the trends and focal points within the scientific discourse on quercetin, capitalizing on the unique informational value offered by author keywords and Keywords Plus.

Before conducting the formal analysis, a data cleansing process was undertaken for both author keywords and Keywords Plus. This process involved the elimination of non‐informative keywords, the standardization of singular and plural forms, and the consolidation of synonyms and abbreviations. Such preprocessing is crucial for refining the dataset and improving the integrity and precision of the analysis. Ultimately, a total of 501 author keywords and 588 Keywords Plus were identified. The terms “quercetin,” “flavonoid,” “antioxidant,” “oxidative stress,” and “cancer” rank among the most prevalent keywords in both the author keywords and the Keywords Plus, underscoring their importance as prominent research focal points within the field of quercetin studies. Additionally, other significant terms such as “apoptosis,” “bioavailability,” “metabolism,” “cardiovascular,” “cell,” “polyphenol,” and “NF‐κB” are also represented among the top 20 high‐frequency keywords in both categories. This suggests that these concepts represent critical and extensively examined facets within the realm of quercetin research, highlighting the complexity and diversity of the subject. In particular, the appearance of “NF‐κB” as a high‐frequency keyword is significant. The presence of “NF‐κB” is indicative of its role in regulating numerous cellular processes, such as inflammation and immune responses, thereby highlighting its significance in this domain. This observation implies that the pharmacological mechanisms through which quercetin exerts therapeutic effects across various diseases, including cancer, immune system disorders, and other conditions such as neurological diseases, are linked to the NF‐κB signaling pathway (Granado‐Serrano et al. 2010; Cheng et al. 2019; Islam et al. 2025). Overall, both author keywords and Keywords Plus demonstrated analogous research topics, hotspots, and trends. Table 5 enumerates the 20 most frequently occurring keywords.

**TABLE 5:** Top 20 keywords with most frequent occurrence.

No.	Author keywords	Occurrence	Keywords plus	Occurrence
1	Quercetin	185	Flavonoid	86
2	Flavonoid	38	Oxidative stress	41
3	Antioxidant	24	Antioxidant	40
4	Oxidative stress	22	Cancer	40
5	Cancer	19	Quercetin	40
6	Apoptosis	15	In vitro	36
7	Rutin	13	Cell	31
8	Bioavailability	12	Inhibition	29
9	Inflammation	11	Cardiovascular	22
10	Metabolism	11	Apoptosis	21
11	Nanoparticle	11	NF‐κB	20
12	Diabetes	10	Absorption	19
13	Cell	8	Metabolism	19
14	Cardiovascular	7	Lipid‐peroxidation	18
15	Kaempferol	7	In‐vivo	17
16	Polyphenol	7	Polyphenol	17
17	Alzheimer's disease	6	Expression	17
18	Free radicals	6	Bioavailability	16
19	Hypertension	6	Glycosides	15
20	NF‐κB	6	Nitric‐oxide	15

**FIGURE 5:**
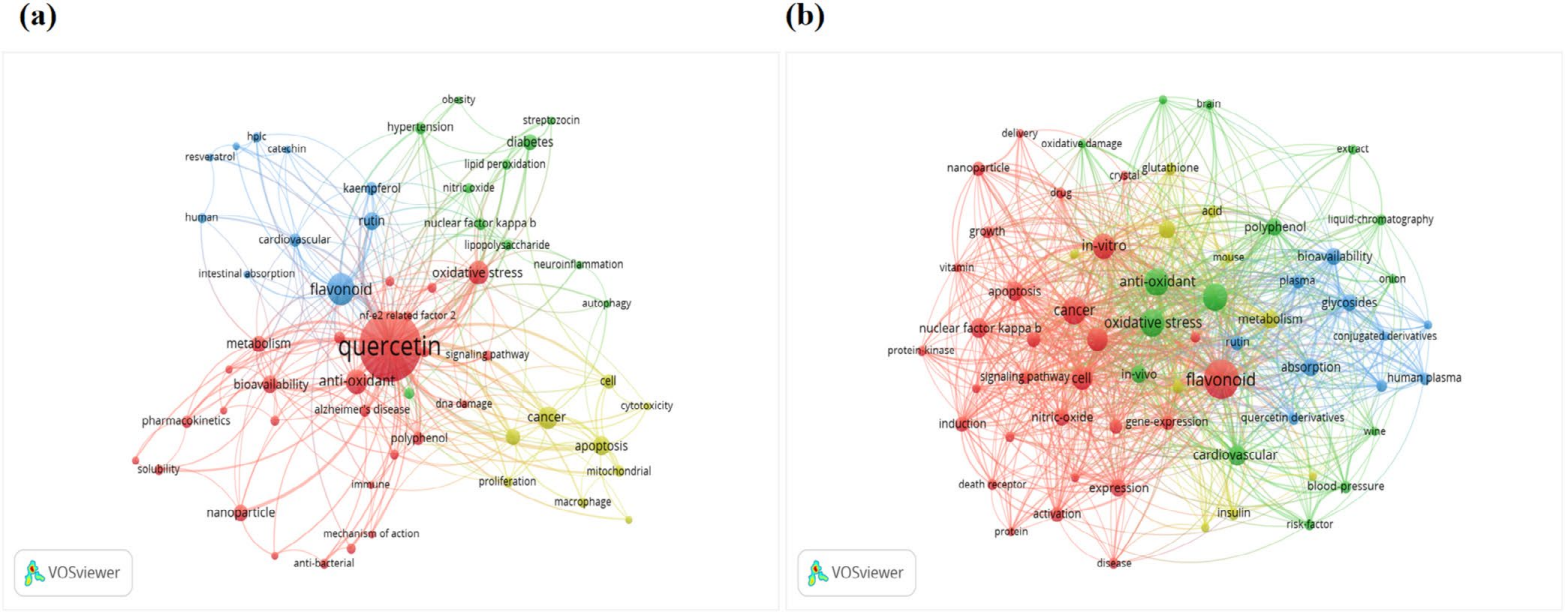
Author keywords (a) and keywords plus (b) co‐occurrence network visualization of the 200 most‐cited articles on quercetin.

Figure 5a,b illustrates the network visualization diagrams for author keywords and Keywords Plus, depicting the co‐occurrence relationships among the keywords. In the analysis of author keywords, only 25 terms appeared more than five times. In contrast, the examination of Keywords Plus revealed that 64 terms exceeded this frequency threshold. This suggests that Keywords Plus encompasses a greater number of high‐frequency keywords likely due to its automatic generation mechanism. This mechanism employs an algorithm to extract keywords from the titles of cited references, thereby identifying a broader array of pertinent terms. In the visualization map of author keywords, only those keywords with a frequency of three or more are presented, resulting in a total of 58 keywords meeting this threshold. While, in the Keywords Plus visualization map, keywords with a frequency of five or more are displayed, yielding a total of 64 keywords that satisfy this threshold. Furthermore, the selected keywords can be broadly divided into four distinct clusters, with each cluster represented by nodes of different colors: red, yellow, green, and blue. Keyword co‐occurrence network analysis revealed distinct four‐color clustering patterns in quercetin research. In the author keywords network: the red cluster (molecular mechanisms and nano‐delivery) contained key terms including oxidative stress and nanoparticles; the blue cluster (inflammatory diseases) focused on diabetes and neuroinflammation; the green cluster (polyphenol composition and analytical techniques) highlighted flavonoids and HPLC, while the yellow cluster (cellular regulatory network) emphasized apoptosis and NF‐κB. The Keywords Plus network exhibited similar four‐color clustering: the red cluster (molecular mechanisms and delivery systems) aggregated terms such as apoptosis and nanoparticles; the blue cluster (cardiovascular and neuroprotection) centered on cardiovascular diseases and Alzheimer's disease; the green cluster (analytical methods and derivatives) included liquid chromatography and quercetin derivatives; and the yellow cluster (metabolic regulation) involved insulin and metabolism. Network analysis demonstrated that the red clusters in both networks consistently focused on molecular mechanisms, blue clusters on disease research, green clusters on methodology, and yellow clusters on regulatory mechanisms. This consistent clustering pattern validates the structural stability of quercetin research knowledge architecture. Notably, strong interconnections between red and blue clusters through key nodes (e.g., NF‐κB) revealed the translational pathway from fundamental mechanisms to clinical applications, while associations between green and red clusters (e.g., bioavailability‐pharmacokinetics) reflected the synergistic development of research methods and technological innovations. This color‐coded dual‐network clustering analysis systematically demonstrates the multidimensional knowledge system of quercetin research and its intrinsic correlations.

### 3.9 Research Topic Analysis

Keywords are typically intimately linked to the central themes and concepts of an article. Keyword analysis is a widely employed technique in academic research capable of elucidating the primary content research focus and developmental trends within a specific field. Through the examination of high‐frequency keywords using common software tools such as VOSviewer and CiteSpace, the interrelationships among keywords are visually represented, thereby facilitating the identification of research topics hotspots and trends within the literature. Nevertheless this method does not consistently encapsulate the core content of research articles with precision. To address this issue, we undertook a comprehensive manual review of each article encompassing the title abstract and full text and subsequently categorized the research topics of 200 most‐cited articles.

**FIGURE 6:**
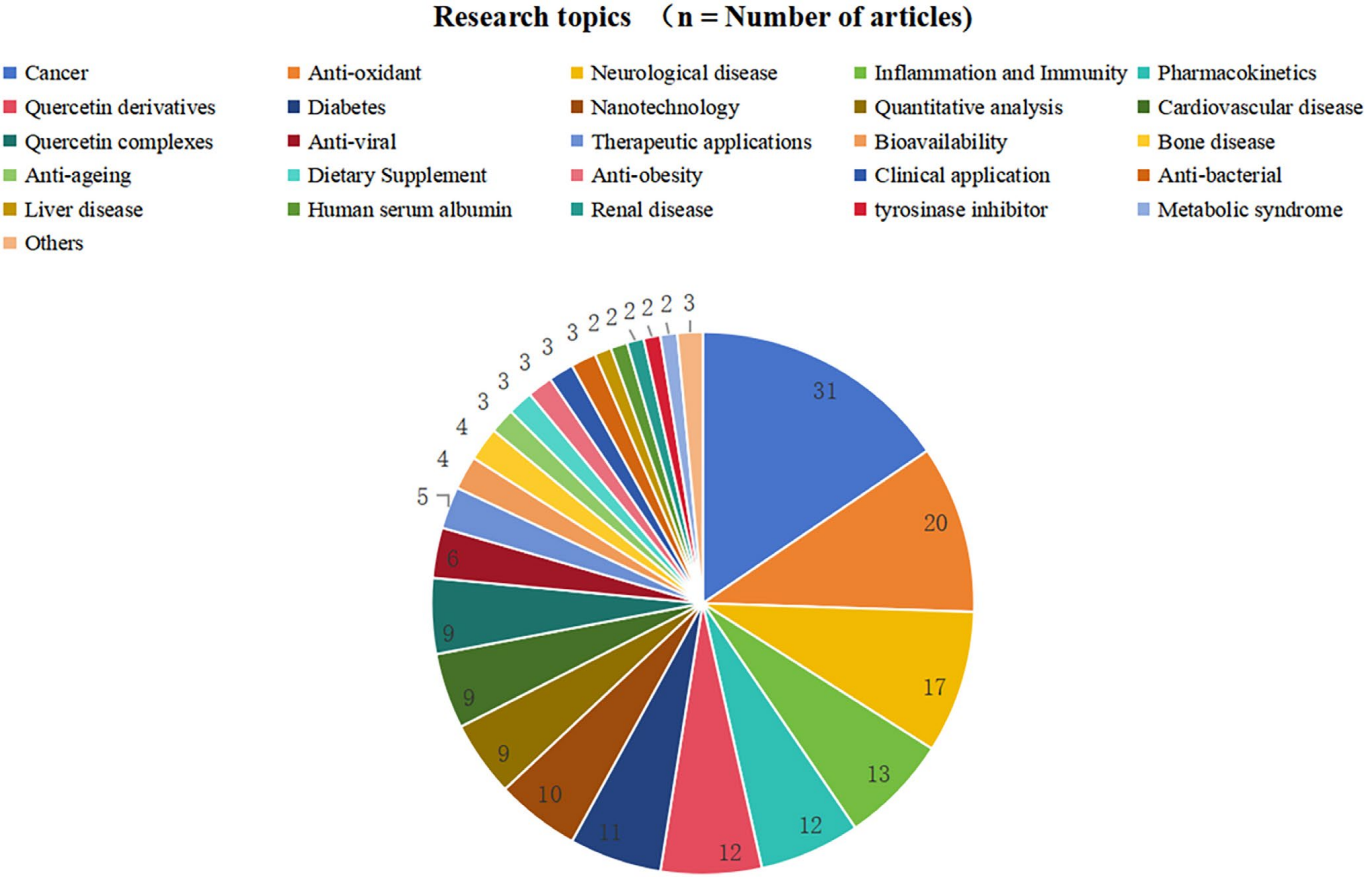
Classification of research topics in the 200 most‐cited quercetin articles.

The 200 most‐cited articles on quercetin were manually evaluated and categorized into over 20 research topic. These include its role in treating various diseases, primary mechanisms of action like antioxidant, anti‐inflammatory, and immunomodulatory effects, pharmacokinetic studies, the use of nanotechnology to enhance bioavailability, stability, and solubility, quantitative and qualitative analyses of quercetin and its derivatives, research on quercetin derivatives, and integrated applications of quercetin. In summary, scholarly articles investigating the role of quercetin in cancer are the most prevalent and have received the highest number of citations (*n* = 31; 7271 citations). These are followed by studies focused on “antioxidant‐related” topics (*n* = 20; 6513 citations), “neurological disease‐related” topics (*n* = 17; 3544 citations), “inflammation and immunity‐related” topics (*n* = 13; 4152 citations), and “pharmacokinetics‐related” topics (*n* = 12; 3334 citations). These research topics represent the domains of quercetin that have attracted the most scholarly attention. The research topics of the 200 most‐cited articles on quercetin have been meticulously categorized, with the corresponding article numbers provided in Table S4. Figure 6 presents the categorization of research topics for the 200 most frequently cited articles on quercetin.

### 3.10 Research Areas Analysis

We conducted a statistical analysis of the research domains of the 200 most‐cited articles on quercetin, revealing that these articles encompassed over 30 distinct research areas. Notably, the fields of “biochemistry and molecular biology” (*n* = 40; 9997 citations), “pharmacology and pharmacy” (*n* = 39; 11,208 citations), “nutrition and dietetics” (*n* = 20; 6268 citations), “agriculture, multidisciplinary” (*n* = 14; 3738 citations), and “chemistry” (*n* = 13; 3203 citations) emerged as the most frequently represented areas in quercetin‐related research.

**FIGURE 7:**
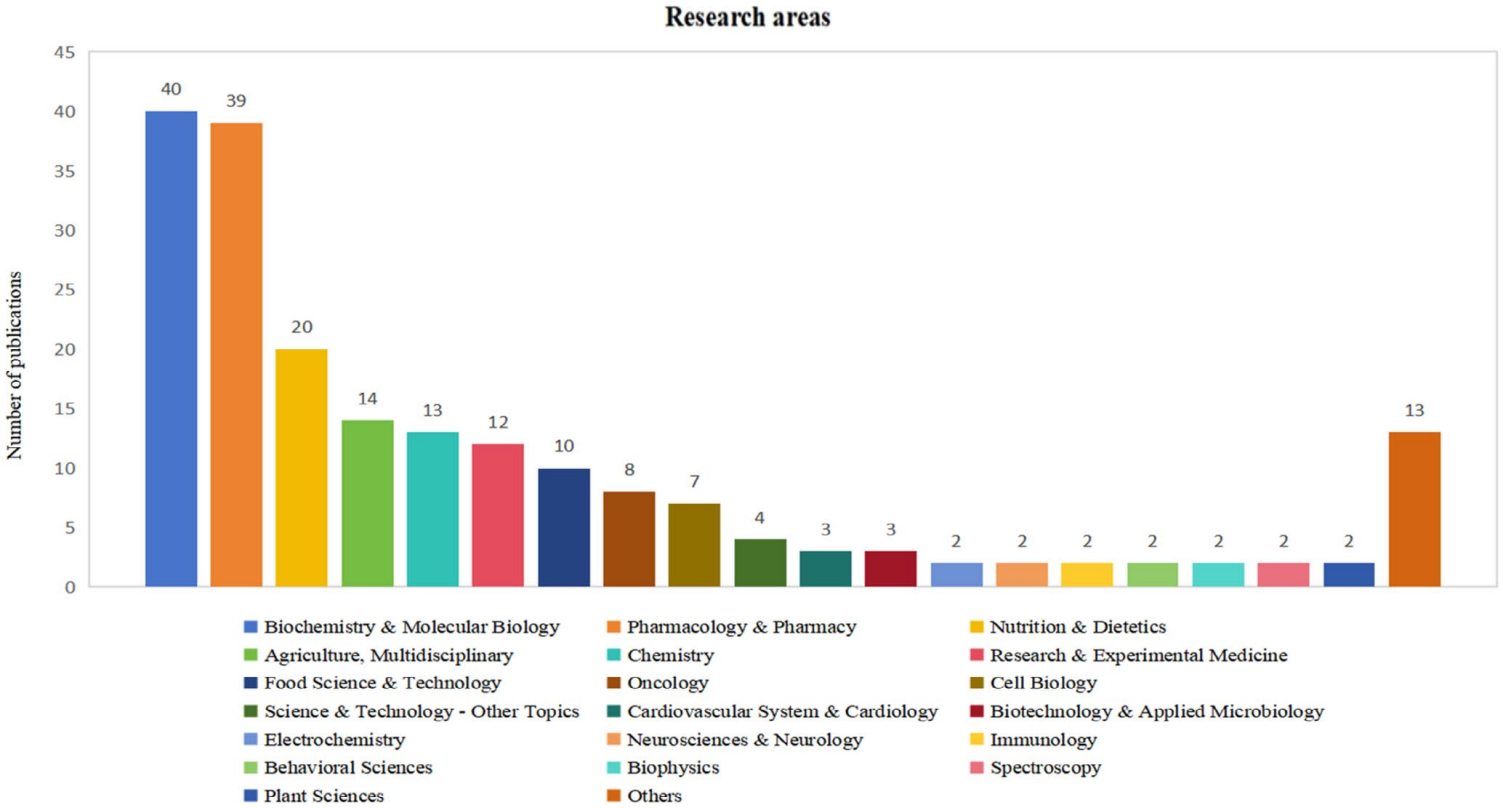
Classification of research areas in the 200 most‐cited quercetin articles.

Overall, a collective analysis of quercetin research domains demonstrates the field's central aim: to establish a systematic framework for understanding its fundamental biological and chemical properties. This includes investigating its mechanisms of action at the molecular and cellular levels, examining its pharmacological effects, and evaluating its potential health benefits. Furthermore, scholarly inquiry also emphasizes the extensive application of quercetin across the pharmaceutical, nutritional, and industrial sectors. The detailed classification of research areas and the corresponding number of articles are provided in Table S5. Figure 7 presents the categorization of research areas for the 200 most frequently cited articles on quercetin.

## 4 Discussion

### 4.1 Current Research Status

This study analyzed 200 most‐cited quercetin articles, offering a systematic perspective on the research landscape through bibliometrics. Among these articles, original research papers constitute 79%, while review articles account for 21%, indicating a preference among researchers for citing primary studies. An analysis of publication years and citation counts reveals two peaks in quercetin research: the first between 2003 and 2011 and the second in 2019. In light of the research trends pertaining to quercetin, it is evident that since the 20th century, quercetin has garnered considerable attention from the scientific community. Researchers have undertaken extensive investigations, resulting in a substantial body of high‐quality and groundbreaking discoveries, notably around the years 2008 and 2019. Approximately, 60% of the most‐cited articles were authored by researchers from Asian countries, with European and North American countries following. Notably, China and the United States have made substantial contributions, leading in both publication output and citation counts. The dominant positions of China and the United States may be attributed to their sustained policy commitments and substantial research funding in the field of natural product studies. Additionally, Tokushima University in Japan, the University of Kiel in Germany, and Shandong University in China are ranked among the top three institutions based on both publication output and citation counts. Bibliometric analysis identifies Wolffram Siegfried and Hollman PCH as the foremost contributors to quercetin research, with their dominance particularly evident in high‐citation publications. This conclusion is corroborated by data from additional databases. Wolffram Siegfried and Hollman PCH have undertaken extensive research on quercetin, encompassing its bioavailability, pharmacokinetics, effects on gene expression, drug interactions, and health benefits across a range of diseases. Their research encompasses the disciplines of nutrition and dietetics, pharmacology, and food science and technology, thereby establishing a scientific basis for the application of quercetin in the prevention and treatment of associated diseases (Egert et al. 2008; Lesser and Wolffram 2006; Dower et al. 2015; Burdeos et al. 2020).

This study encompasses an analysis of 115 journals. Based on publication output and citation counts, 12 journals have been identified as the principal platforms for the dissemination of research related to quercetin (Table S7). Among these, the *Journal of Agricultural and Food Chemistry* is distinguished by its leading position in both publication volume and citation frequency. It has maintained an impact factor of 6 over the past 5 years and holds a Q1 ranking in the JCR, underscoring its sustained prominence and influence in this research domain. Based on the impact factor rankings, the *Journal of Hepatology* holds the highest impact factor at 28, followed by *Autophagy* with an impact factor of 16.8, and *Trends in Food Science & Technology* at 16.4. Each of these journals has published one paper. In Yao et al. 2007, Yao et al. from Germany, published an article entitled “Quercetin Protects Human Hepatocytes from Ethanol‐Derived Oxidative Stress by Inducing Heme Oxygenase‐1 via the MAPK/Nrf2 Pathways” in the *Journal of Hepatology*, which has been cited 314 times. In Wang et al. 2011, Wang et al. from China published an article entitled “Quercetin Induces Protective Autophagy in Gastric Cancer Cells: Involvement of Akt‐mTOR‐ and Hypoxia‐Induced Factor 1α‐Mediated Signaling” in the journal *Autophagy*, which has been cited 323 times. Subsequently, in Wang et al. 2016, Wang and et al. from China published a comprehensive review titled “The Biological Activities, Chemical Stability, Metabolism, and Delivery Systems of Quercetin” in *Trends in Food Science & Technology*, which has garnered 514 citations. These three articles are highly cited, consistently appearing within the top 50 for citation frequency (Yao et al. 2007; Wang et al. 2011, 2016). They offer an in‐depth exploration of the diverse biological activities of quercetin from multiple perspectives, including its hepatoprotective effects, anticancer potential, and the innovative application of quercetin in cancer treatment via nanotechnology.

### 4.2 Research Hotspots and Topic

Based on the findings from the keyword co‐occurrence analysis, it was noted that terms such as “antioxidant,” “oxidative stress,” “cancer,” “apoptosis,” “bioavailability,” “metabolism,” “cell,” and “NF‐κB” frequently appeared among both the author keywords and Keyword Plus. Through integrated analysis of high‐frequency terms and co‐occurrence networks from both author keywords and Keywords Plus, this study identifies the following core research hotspots in quercetin studies: (1) “Molecular mechanisms and delivery systems”–investigations primarily focus on oxidative stress (Xu et al. 2019) (total frequency: 63) and NF‐κB signaling pathways (26), while advancements in nanoparticle delivery technology (11), particularly through liposomal and polymeric nanoparticle systems, have significantly enhanced bioavailability (28) (Cai et al. 2013); (2) “Therapeutic applications” (Okamoto 2005; Bischoff 2008)–quercetin demonstrates potential in managing cardiovascular diseases (29), Alzheimer's disease (6), diabetes (10), and cancer (59), with its therapeutic effects attributed to multifaceted bioactivities including anti‐inflammatory, antioxidant, and immunomodulatory properties; (3) “Active constituents and analytical methods”–flavonoid compounds (124) and their glycosylated derivatives (glycosides, 15) represent primary research targets, with the predominant occurrence of “flavonoid” (ranked first) underscoring quercetin's central position among flavonoids, while technological progress in HPLC‐MS coupling has enabled reliable qualitative and quantitative analysis of quercetin and its metabolites (19) (Wach et al. 2007; Moon et al. 2008; Ishisaka et al. 2011); (4) “Metabolic and cellular regulation”‐ studies elucidate quercetin's effects on apoptosis (51), autophagy (15), and metabolic regulation (30), establishing a multi‐level regulatory network from molecular mechanisms to systemic physiological effects. These regulatory mechanisms enable quercetin to exert significant therapeutic effects in various diseases, particularly in cancer by selectively inducing apoptosis across multiple malignancies (including breast, colon, and hematologic cancers) as demonstrated in vitro and in vivo (Hashemzaei et al. 2017) and in osteoarthritis through mitigating oxidative stress‐induced chondrocyte apoptosis (Feng et al. 2019). Crucially, these domains form an interconnected knowledge network through key nodes (e.g., NF‐κB), demonstrating remarkable translational medicine characteristics where fundamental discoveries inform clinical applications, and technological innovations (e.g., delivery system optimization and analytical method improvements) reciprocally enhance mechanistic investigations, thereby establishing a virtuous “basic‐translational‐applied” research cycle that solidifies the foundation for quercetin's further development and application.

Notably, among the 200 most‐cited publications, research on quercetin purification technologies accounts for a relatively small proportion. However, as a critical technical prerequisite for industrialization and commercialization, this field remains of significant research value. Literature analysis indicates that quercetin is primarily extracted and purified from natural matrices using organic solvent systems (Srinivas et al. 2010), with extraction efficiency being significantly influenced by parameters including solvent polarity, temperature, and pH. Given that quercetin predominantly exists in glycosylated forms in nature, its purification process further requires addressing the key technical challenge of selective glycoside hydrolysis. The primary research challenges currently lie in effectively improving purification efficiency and product yield, which urgently demands the development of novel green extraction technologies and systematic scale‐up studies to advance quercetin's industrial and commercial application processes. Overall, these hotspots provide a comprehensive overview, illustrating diverse facets of quercetin research, ranging from its bioactivity and associations with diseases to the underlying molecular mechanisms and the research methods and techniques employed. Notably, this study demonstrates high methodological consistency with the bibliometric analysis of curcumin research (Zhou et al. 2022). Both investigations employed VOSviewer software to conduct co‐occurrence analysis and construct knowledge mapping networks using the highly cited literature. The results reveal that quercetin and curcumin, as two important natural bioactive compounds, share core research focuses including biological activities (anti‐inflammatory, antioxidant, and anticancer effects), molecular mechanisms (e.g., NF‐κB signaling pathway regulation), and bioavailability. These findings not only validate the applicability of bibliometric approaches in natural product research but, more significantly, through comparative analysis, uncover potential common research paradigms among different bioactive natural compounds. The bibliometric analysis based on highly cited literature provides a robust methodological framework for systematically understanding research trends in natural products.

An analysis of the content of each article reveals that research on quercetin encompasses a broad spectrum of topics, primarily including those related to “cancer,” “antioxidant,” “inflammation and immunity,” “neurological diseases,” and “pharmacokinetics.” Notably, the body of research concerning cancer is the most extensive. A review of over 30 articles pertaining to quercetin and cancer research indicates that quercetin holds significant potential in the prevention and treatment of tumors and cancers. Quercetin has demonstrated potential anti‐cancer properties by inhibiting cancer cell proliferation, inducing apoptosis and autophagy, and suppressing angiogenesis and metastasis through modulation of various signal transduction pathways, including MEK/ERK (Murakami et al. 2008), Nrf2/Keap1 (Tanigawa et al. 2007), PI3K/AKT/mTOR (Granato et al. 2017), and Wnt/β‐catenin (Srivastava and Srivastava 2019). These effects have been observed across multiple cancer cell lines and animal models, encompassing breast, lung, colorectal, prostate, ovarian, and gastric cancers. In research on other topics, quercetin has been extensively investigated by researchers to elucidate its mechanisms of action and pharmacological effects in disease treatment and prevention. These investigations have highlighted its diverse properties, including antioxidant, anti‐inflammatory, anti‐allergic, antiviral, anti‐aging, anti‐obesity, antibacterial, and anti‐angiogenic activities. Furthermore, quercetin has shown potential in the amelioration and treatment of disorders affecting the nervous, cardiovascular, hepatic, renal, and skeletal systems. The objective of pharmacokinetic research is to elucidate the processes of absorption, distribution, metabolism, and excretion of quercetin, including its metabolites and derivatives, within human and animal systems as well as to examine its interactions with other pharmaceuticals. Concurrently, research in the domain of nanotechnology aims to improve the stability, solubility, and bioavailability of quercetin, while extending its half‐life and minimizing its toxicity. These investigations also explore the integration of quercetin with delivery materials to facilitate precision therapy for tumors and cancerous diseases.

The research areas of the 200 most‐cited articles were predominantly concentrated in “biochemistry and molecular biology” and “pharmacology and pharmacy.” Additionally, they encompassed interdisciplinary and multidisciplinary research, exemplified by the integration of “pharmacology and pharmacy” with “chemistry,” as well as the convergence of “food science and technology” with “nutrition and dietetics.” This suggests that research on quercetin is highly esteemed not only within the conventional biomedical field but also gains from the synergistic benefits of interdisciplinary and multidisciplinary methodologies. These collaborative research initiatives are enhancing the scientific community's comprehensive understanding and application of this natural compound. Moreover, the trend toward integrated interdisciplinary and multidisciplinary research may facilitate the development of innovative applications and drug formulations involving quercetin.

### 4.3 Research Opportunities and Challenges

Extensive research has established quercetin's multi‐target mechanisms and therapeutic potential, particularly in antioxidant, anti‐inflammatory, anti‐cancer, cardiovascular, and neuroprotective applications for disease prevention and treatment. Notably, numerous studies suggest that quercetin may function as a modulator of multidrug resistance (MDR) and serve as a potential chemosensitizer (Chen et al. 2010). Quercetin and its derivatives have evolved beyond their traditional role as dietary components, emerging as promising candidates in drug development initiatives (Patel et al. 2018). Owing to their diverse biological activities and favorable safety profile, these compounds hold significant potential for development into novel pharmaceuticals or as adjunctive agents to existing treatments. Therefore, quercetin presents a natural and safe alternative for the creation of new therapeutic drugs. Its abundant availability and ease of access further enhance its potential utility in the formulation of food supplements and pharmaceutical products. Moreover, the antioxidant, anti‐aging, and immunomodulatory properties of quercetin suggest its potential utility as an ingredient in anti‐aging and skin protection formulations within the cosmetics industry (Chondrogianni et al. 2010). Comprehensive pharmacokinetic studies are essential for optimizing dosage design, thereby enhancing quercetin's bioavailability and therapeutic efficacy (Ulusoy and Sanlier 2020). The application of nanotechnology offers innovative solutions for improving quercetin's drug delivery systems. Research on quercetin spans multiple disciplines, including pharmacology, molecular biology, and chemistry, thereby providing substantial opportunities for interdisciplinary exploration.

Nevertheless, the investigation of quercetin presents several challenges. A primary concern is its inherently low bioavailability, attributed to its rapid metabolism and excretion in vivo, which consequently diminishes its bioactivity and constrains its therapeutic potential (Wang et al. 2016) The bioavailability of quercetin is subject to considerable inter‐individual variability, necessitating further research to elucidate whether this variability is associated with factors such as dietary history, food matrix, disease status, genetic polymorphisms, and gut microbiome metabolism (Almeida et al. 2018). Quercetin has the potential to interact with other medications or nutrients, which may influence its therapeutic efficacy and safety profile. Furthermore, the long‐term safety and possible adverse reactions associated with quercetin are not fully understood, necessitating further research for a holistic evaluation. The diverse origins of quercetin present challenges in standardization and quality control that must be addressed within quercetin research. Despite the substantial body of research on quercetin, it remains predominantly accessible as an over‐the‐counter dietary supplement (Andres et al. 2018). Consequently, there is a pressing need for further investigation into its stability, palatability, and nutritional value across diverse food systems. Furthermore, it is essential to enhance the development of industry regulations and standards governing the use of quercetin as a dietary supplement to ensure its safety and efficacy. Despite quercetin demonstrating significant efficacy in vitro and in animal studies, thereby providing promising preliminary evidence, there remains a substantial lack of clinical research on quercetin (Hosseini et al. 2021). Among the 200 most‐cited articles, only three pertain to clinical investigations (Egert et al. 2009; Okamoto 2005; Dower et al. 2015); of these, one is a review evaluating the clinical safety of quercetin, while the other two are randomized, double‐blind, placebo‐controlled crossover trials focused on cardiovascular diseases. This suggests that the current body of research regarding the clinical effects and safety of quercetin in humans remains inadequate, necessitating further clinical trials to substantiate its efficacy in the treatment of various diseases. Research on quercetin has reached a relatively advanced stage in specific disease areas, particularly within oncology, cardiovascular diseases, and neurodegenerative disorders. Investigations in these domains have not only illuminated the potential therapeutic effects of quercetin but also established a foundation for future clinical applications. However, the mechanisms and effects of quercetin across a range of disease states, such as autoimmune diseases, metabolic disorders, osteoporosis, osteoarthritis, pulmonary diseases, diabetes, aging, mood disorders, gout, and hyperuricemia, necessitate further investigation to completely elucidate its therapeutic potential as a natural compound.

Further, in‐depth exploration is required in the research on quercetin, particularly in the following areas: the identification of specific molecular targets and mechanisms of action in various diseases, with an emphasis on rare and uncommon conditions; the pharmacokinetics and pharmacodynamics of quercetin; the optimization of dosage and dietary intake; determining the appropriate dosage and form for disease prevention; the development of personalized therapeutic approaches; understanding biological barriers, such as blood–brain barrier permeability; the regulation of cancer MDR; the efficacy of combined treatment modalities that incorporate quercetin with other therapeutic approaches; the development of novel food‐grade delivery systems to enhance the bioavailability, targeting, and safety of quercetin; the investigation of structure–activity relationships of quercetin and its derivatives; and the refinement of quantitative analytical methods and techniques for quercetin assessment.

In recent years, the quercetin research field has demonstrated significant thematic evolution. Bibliometric analysis reveals that traditional research focuses such as antioxidant and anti‐inflammatory effects have shown declining research interest, while emerging directions including nano‐delivery systems (Sadalage et al. 2021; Zhao et al. 2025), ferroptosis regulation mechanisms (Li et al. 2024; Xiong et al. 2025), and interdisciplinary applications like the gut‐brain axis (Balasubramanian et al. 2023; Wu et al. 2024), microbiome remodeling (Zhao et al. 2021), and epigenetic modulation (Shi et al. 2025) are garnering increasing attention. This shift reflects a transition from fundamental activity evaluation and pharmacological mechanism studies toward more in‐depth investigations of bioavailability enhancement, precise mechanism exploration, and interdisciplinary integration. Such evolution in research priorities not only demonstrates the continued vitality of quercetin research but also provides new directions for future clinical applications and mechanistic studies. In conclusion, the domain of quercetin research presents numerous opportunities; however, it must address a range of scientific and technological challenges to facilitate its extensive application in the realms of medicine, food, and cosmetics. These research trajectories are essential for a comprehensive understanding of the biological activity and application potential of quercetin, thereby establishing a scientific foundation for its utilization in the fields of medicine, food, and nutrition. It is anticipated that quercetin holds significant potential for future applications across a range of domains, including personalized therapy, combination therapy, novel drug development, preventive healthcare, and interdisciplinary research.

## 5 Conclusions

This bibliometric analysis offers a multidimensional visual representation of the 200 most‐cited academic articles on quercetin published between 2000 and 2023. These articles are predominantly original research papers. China and the United States are the major contributors to the field of quercetin research. Tokushima University (Japan), Kiel University (Germany), and Shandong University (China) stand out as the most active publishing institutions in this research area. Wolfram Siegfried and Hollman PCH are widely recognized as leading contributors in the field. The *Journal of Agricultural and Food Chemistry* is considered the most distinguished journal in this field. Through keyword analysis, we identified that “molecular mechanisms and delivery systems,” “therapeutic applications,” “active constituents and analytical methods”, and “metabolic and cellular regulation” are the prominent hotspots within the field of quercetin research. Our extensive analysis suggests that the primary research topics in quercetin research include its anti‐cancer, antioxidant, and anti‐inflammatory properties, alongside its functions in immune modulation, neurological disease treatment, pharmacokinetics, and bioavailability. The field of quercetin research still faces several unresolved issues and challenges that necessitate further investigation. These issues and challenges include its bioavailability, long‐term safety and potential side effects, its applications as dietary supplement, the need for in‐depth clinical studies, the elucidation of specific mechanisms of disease treatment, and the refinement of research techniques and methodologies. The quercetin research landscape has shifted from traditional antioxidant studies toward cutting‐edge investigations of nano‐delivery systems, ferroptosis regulation, gut‐brain axis interactions, microbiome modulation, and epigenetic control, reflecting its expanding therapeutic potential. In this study, the basic information about the quercetin research field, including basic information of highly cited articles, countries, institutions, journals, and authors, as well as research hotspots, themes, and directions were summarized and discussed in depth through quantitative analysis methods.

### 5.1 Limitation

This approach offers researchers a deeper understanding, especially in revealing the content and trends of research hotspots. Nonetheless, our study is not without its limitations. First, our literature search was limited to the WoSCC database and may have missed relevant literature from other databases. Second, our inclusion criteria were restricted to articles and reviews published in English, which may have led to the exclusion of non‐English publications, resulting in publication bias. Moreover, given that citation peaks typically occur within 2–6 years post‐publication (Min et al. 2021), our analysis may not adequately reflect the impact of recently published articles. As a result, some recently published but promising articles may not receive sufficient recognition due to insufficient citation accumulation. Despite these limitations, our study provides a systematic overview of research hotspots, topics, and areas related to quercetin, providing valuable insights for current and future research efforts.

## Author Contributions

**Aijuan Yang:** conceptualization (lead), data curation (lead), formal analysis (equal), methodology (lead), resources (equal), software (equal), writing – original draft (lead). **Jiyun Wang:** conceptualization (equal), data curation (equal), formal analysis (lead), methodology (equal), resources (equal), writing – original draft (equal). **Wei Yang:** conceptualization (equal), data curation (equal), formal analysis (equal), methodology (equal), software (lead). **Zhengfei Yang:** conceptualization (equal), resources (lead), validation (equal), visualization (equal), writing – review and editing (equal). **Liming Zhang:** methodology (equal), resources (equal), supervision (lead), visualization (equal), writing – review and editing (equal). **Hangying Li:** conceptualization (equal), data curation (equal), formal analysis (equal), methodology (equal), writing – review and editing (lead).

## Conflicts of Interest

The authors declare no conflicts of interest.

## Supporting information


Appendix S1.

## Data Availability

The data that support the findings of this study are available in the Supporting Information of this article. The data that support the findings of this study are available from the corresponding author upon reasonable request.
