# Supplementary material for: Bibliometric Mapping of Quercetin Research: Analysis of the Most‐Cited Articles (2000–2023)
Source: Food Sci Nutr. 2025 Jul 10;13(7):e70500. doi: 10.1002/fsn3.70500 (PMC12245725; doi:10.1002/fsn3.70500)
Supplement: Supplementary file 1 — Appendix S1. [file FSN3-13-e70500-s001.docx]

Appendices（Supplementary Material）

### Final Search Strategy

Database: Web of Science Core Collection (SCI-Expanded)
Time Span: 2000-01-01 to 2023-12-31
Search Date: September 24, 2024
Query: TI=(“Quercetin”) AND AB=(“Quercetin”) AND AK=(“Quercetin”) AND LANGUAGE:(“English”) AND DOCUMENT TYPES:(“Article” OR “Review”)

NOT DOCUMENT TYPES:(“Retracted Publication” OR “Proceedings Paper” OR “Editorial Material” OR “Letter”OR “Book Chapter” OR “Early Access”)

**TABLE S1** Basic characteristics of the 200 most cited articles on the topic. (page 2-32)

**TABLE S2** Authors of the 200 most-cited articles on Quercetin (number of articles ≥ 3). (page 32)

**TABLE S3** The basic information of the major journals. (page 33)

**TABLE S4** Classification of research topics in the 200 most-cited quercetin articles. (page 33-36)

**TABLE S5** Classification of research areas in the 200 most-cited quercetin articles. (page 36-39)

**FIGURE S1:** Trends of quercetin in scientific research. (page 40)

**FIGURE S2:** The discipline categories of quercetin research. (page 41)

| **TABLE S1** Basic characteristics of the 200 most cited articles on the topic | | | | | | | |
| --- | --- | --- | --- | --- | --- | --- | --- |
| **NO.** | **Article Title** | **First Authors** | **Type** | **Journal(IF)** | **Year** | **Citations** | **ACY*** |
| 1 | Health effects of quercetin: From antioxidant to nutraceutical | Boots, Agnes W. | Review | European Journal of Pharmacology(4.3) | 2008 | 1409 | 82.88 |
| 2 | Quercetin, Inflammation and Immunity | Li, Yao | Review | Nutrients(5.8) | 2016 | 1068 | 118.67 |
| 3 | Flavonoid (myricetin, quercetin, kaempferol, luteolin, and apigenin) content of edible tropical plants | Miean, KH | Article | Journal of Agricultural and Food Chemistry(6) | 2001 | 905 | 37.71 |
| 4 | Antioxidant Activities of Quercetin and Its Complexes for Medicinal Application | Xu, Dong | Review | Molecules(4.6) | 2019 | 733 | 122.17 |
| 5 | Senolytics decrease senescent cells in humans: Preliminary report from a clinical trial of Dasatinib plus Quercetin in individuals with diabetic kidney disease | Hickson, LaTonya J. | Article | Ebiomedicine(9.2) | 2019 | 700 | 116.67 |
| 6 | Review of the flavonoids quercetin, hesperetin naringenin. Dietary sources, bioactivities, and epidemiology | Erlund, I | Review | Nutrition Research(3.5) | 2004 | 696 | 33.14 |
| 7 | Quercetin, a flavonoid antioxidant, prevents and protects streptozotocin-induced oxidative stress and β-cell damage in rat pancreas | Coskun, O | Article | Pharmacological Research(9) | 2005 | 592 | 29.6 |
| 8 | Multitargeted cancer prevention by quercetin | Murakami, Akira | Review | Cancer Letters(8.3) | 2008 | 560 | 32.94 |
| 9 | Quercetin: A flavonol with multifaceted therapeutic applications? | D'Andrea, Gabriele | Review | Fitoterapia(2.7) | 2015 | 554 | 55.4 |
| 10 | Antioxidant and anti-inflammatory activities of quercetin and its derivatives | Lesjak, Marija | Article | Journal of Functional Foods(4.6) | 2018 | 552 | 78.86 |
| 11 | Anti-inflammatory properties of plant flavonoids. Effects of rutin, quercetin and hesperidin on adjuvant arthritis in rat | Guardia, T | Article | Farmaco(-) | 2001 | 535 | 22.29 |
| 12 | The flavonoid quercetin in disease prevention and therapy: Facts and fancies | Russo, Maria | Article | Biochemical Pharmacology(5.2) | 2012 | 529 | 40.69 |
| 13 | Enhancement of gastrointestinal absorption of quercetin by solid lipid nanoparticles | Li, HouLi | Article | Journal of Controlled Release(10.6) | 2009 | 520 | 32.5 |
| 14 | The biological activities, chemical stability, metabolism and delivery systems of quercetin: A review | Wang, Weiyou | Review | Trends in Food Science & Technology(16.4) | 2016 | 514 | 57.11 |
| 15 | Dietary Quercetin and Kaempferol: Bioavailability and Potential Cardiovascular-Related Bioactivity in Humans | Dabeek, Wijdan M. | Review | Nutrients(5.8) | 2019 | 448 | 74.67 |
| 16 | Antidiabetic effects of quercetin in streptozocin-induced diabetic rats | Vessal, M | Article | Comparative Biochemistry and Physiology C-Toxicology & Pharmacology(3.9) | 2003 | 446 | 20.27 |
| 17 | Anticancer potential of quercetin: A comprehensive review | Rauf, Abdur | Review | Phytotherapy Research(7) | 2018 | 428 | 61.14 |
| 18 | Quercetin reduces systolic blood pressure and plasma oxidised low-density lipoprotein concentrations in overweight subjects with a high-cardiovascular disease risk phenotype: a double-blinded, placebo-controlled cross-over study | Egert, Sarah | Article | British Journal of Nutrition(3.5) | 2009 | 421 | 26.31 |
| 19 | Tissue distribution of quercetin in rats and pigs | de Boer, VCJ | Article | Journal of Nutrition(4.3) | 2005 | 412 | 20.6 |
| 20 | Pharmacokinetics of quercetin from quercetin aglycone and rutin in healthy volunteers | Erlund, I | Article | European Journal of Clinical Pharmacology(2.5) | 2000 | 411 | 16.44 |
| 21 | Antioxidative flavonoid quercetin: implication of its intestinal absorption and metabolism | Murota, K | Review | Archives of Biochemistry and Biophysics(3.8) | 2003 | 410 | 18.64 |
| 22 | The anti-inflammatory flavones quercetin and kaempferol cause inhibition of inducible nitric oxide synthase, cyclooxygenase-2 and reactive C-protein, and down-regulation of the nuclear factor kappaB pathway in Chang Liver cells | Garcia-Mediavilla, Victoria | Article | European Journal of Pharmacology(4.3) | 2007 | 409 | 22.72 |
| 23 | The anti-obesity effect of quercetin is mediated by the AMPK and MAPK signaling pathways | Ahn, Jiyun | Article | Biochemical and Biophysical Research Communications(2.7) | 2008 | 403 | 23.71 |
| 24 | A novel electro analytical nanosensor based on graphene oxide/silver nanoparticles for simultaneous determination of quercetin and morin | Yola, Mehmet Lutfi | Article | Electrochimica Acta(5.5) | 2014 | 401 | 36.45 |
| 25 | Iron chelation by the powerful antioxidant flavonoid quercetin | Leopoldini, Monica | Article | Journal of Agricultural and Food Chemistry(6) | 2006 | 389 | 20.47 |
| 26 | Action of Nrf2 and Keap1 in ARE-mediated *NQO1* expression by quercetin | Tanigawa, Shunsuke | Article | Free Radical Biology and Medicine(7.9) | 2007 | 387 | 21.5 |
| 27 | The Anti-Cancer Effect of Quercetin: Molecular Implications in Cancer Metabolism | Reyes-Farias, Marjorie | Review | International Journal of Molecular Sciences(5.6) | 2019 | 382 | 63.67 |
| 28 | Chlorogenic acid, quercetin-3-rutinoside and black tea phenols are extensively metabolized in humans | Olthof, MR | Article | Journal of Nutrition(4.3) | 2003 | 379 | 17.23 |
| 29 | Quercetin: potentials in the prevention and therapy of disease | Bischoff, Stephan C. | Article | Ccurrent Opinion in Clinical Nutrition and Metabolic Care(3.7) | 2008 | 376 | 22.12 |
| 30 | The Pharmacological Activity, Biochemical Properties, and Pharmacokinetics of the Major Natural Polyphenolic Flavonoid: Quercetin | Batiha, Gaber El-Saber | Review | Foods(5.1) | 2020 | 369 | 73.8 |
| 31 | Protective effect of quercetin on high-fat diet-induced non-alcoholic fatty liver disease in mice is mediated by modulating intestinal microbiota imbalance and related gut-liver axis activation | Porras, David | Article | Free Radical Biology and Medicine(7.9) | 2017 | 360 | 45 |
| 32 | Pharmacological basis and new insights of quercetin action in respect to its anti-cancer effects | Tang, Si-Min | Review | Biomedicine & Pharmacotherapy(6.8) | 2020 | 357 | 71.4 |
| 33 | Antihypertensive effects of the flavonoid quercetin in spontaneously hypertensive rats | Duarte, J | Article | British Journal of Pharmacology(7.4) | 2001 | 356 | 14.83 |
| 34 | Therapeutic potential of quercetin as a cardiovascular agent | Patel, Rahul V. | Review | European Journal of Medicinal Chemistry(6.1) | 2018 | 341 | 48.71 |
| 35 | Development of biodegradable nanoparticles for delivery of quercetin | Kumari, Avnesh | Article | Colloids and Surfaces B-Biointerfaces(5.1) | 2010 | 340 | 22.67 |
| 36 | Safety Aspects of the Use of Quercetin as a Dietary Supplement | Andres, Susanne | Review | Molecular Nutrition & Food Research(5.6) | 2018 | 335 | 47.86 |
| 37 | Quercetin alleviates acute kidney injury by inhibiting ferroptosis | Wang, Yue | Article | Journal of Advanced Research(11) | 2021 | 331 | 82.75 |
| 38 | Quercetin and Vitamin C: An Experimental, Synergistic Therapy for the Prevention and Treatment of SARS-CoV-2 Related Disease (COVID-19) | Colunga Biancatelli, Ruben Manuel Luciano | Review | Frontiers in Immunology(4.3) | 2020 | 330 | 66 |
| 39 | Protective effect of quercetin in primary neurons against Aβ(1-42): relevance to Alzheimer's disease | Ansari, Mubeen Ahmad | Article | Journal of Nutritional Biochemistry(5.4) | 2009 | 328 | 20.5 |
| 40 | Induction of cell cycle arrest and apoptosis in human breast cancer cells by quercetin | Choi, JA | Article | International Journal of Oncology(4.6) | 2001 | 328 | 13.67 |
| 41 | Cocrystals of Quercetin with Improved Solubility and Oral Bioavailability | Smith, Adam J. | Article | Molecular Pharmaceutics(4.6) | 2011 | 323 | 23.07 |
| 42 | Quercetin induces protective autophagy in gastric cancer cells Involvement of Akt-mTOR- and hypoxia-induced factor 1α-mediated signaling | Wang, Kui | Article | Autophagy(16.8) | 2011 | 323 | 23.07 |
| 43 | Anticancer and apoptosis-inducing effects of quercetin *in vitro* and *in vivo* | Hashemzaei, Mahmoud | Article | Oncology Reports(3.4) | 2017 | 322 | 40.25 |
| 44 | Absorption, excretion and metabolite profiling of methyl-, glucuronyl-, glucosyl- and sulpho-conjugates of quercetin in human plasma and urine after ingestion of onions | Mullen, William | Article | British Journal of Nutrition(3.5) | 2006 | 321 | 16.89 |
| 45 | Probing the binding of the flavonoid, quercetin to human serum albumin by circular dichroism, electronic absorption spectroscopy and molecular modelling methods | Zsila, F | Article | Biochemical Pharmacology(5.2) | 2003 | 319 | 14.5 |
| 46 | The flavonoid quercetin ameliorates Alzheimer's disease pathology and protects cognitive and emotional function in aged triple transgenic Alzheimer's disease model mice | Maria Sabogal-Guaqueta, Angelica | Article | Neuropharmacology(4.5) | 2015 | 315 | 31.5 |
| 47 | Quercetin protects human hepatocytes from ethanol-derived oxidative stress by inducing heme oxygenase-1 via the MAPK/Nrf2 pathways | Yao, Ping | Article | Journal of Hepatology(28) | 2007 | 314 | 17.44 |
| 48 | Anti-inflammatory, anti-proliferative and anti-atherosclerotic effects of quercetin in human *in vitro* and *in vivo* models | Kleemann, Robert | Article | Atherosclerosis(5.3) | 2011 | 300 | 21.43 |
| 49 | Study of freeze-dried quercetin-cyclodextrin binary systems by DSC, FT-IR, X-ray diffraction and SEM analysis | Pralhad, T | Article | Journal of Pharmaceutical and Biomedical Analysis(3.1) | 2004 | 297 | 14.14 |
| 50 | Bioavailability of Quercetin: Problems and Promises | Cai, X. | Article | Current Medicinal Chemistry(4) | 2013 | 285 | 23.75 |
| 51 | Preparation, physicochernical characterization, and antioxidant effects of quercetin nanoparticles | Wu, Tzu-Hui | Article | International Journal of Pharmaceutics(5.6) | 2008 | 285 | 16.76 |
| 52 | Quercetin, a potent inhibitor against β-catenin/Tcf signaling in SW480 colon cancer cells | Park, CH | Article | Biochemical and Biophysical Research Communications(2.7) | 2005 | 285 | 14.25 |
| 53 | Synthesis, characterization and antioxidant activity copper-quercetin complex | Bukhari, S. Birjees | Article | Spectrochimica Acta Part A-Molecular and Biomolecular Spectroscopy(3.8) | 2009 | 281 | 17.56 |
| 54 | Neuroprotective Effects of Quercetin in Alzheimer's Disease | Khan, Haroon | Review | Biomolecules(5.4) | 2020 | 273 | 54.6 |
| 55 | Quercetin and Its Anti-Allergic Immune Response | Mlcek, Jiri | Review | Molecules(4.6) | 2016 | 272 | 30.22 |
| 56 | Quercetin suppresses proinflammatory cytokines production through MAP kinases and NF-κB pathway in lipopolysaccharide-stimulated macrophage | Cho, SY | Article | Molecular and Cellular Biochemistry(3.3) | 2003 | 271 | 12.32 |
| 57 | Life or death: Neuroprotective and anticancer effects of quercetin | Dajas, Federico | Review | Journal of Ethnopharmacology(4.8) | 2012 | 267 | 20.54 |
| 58 | Quercetin as an Antiviral Agent Inhibits Influenza A Virus (IAV) Entry | Wu, Wenjiao | Article | Viruses-Basel(4) | 2016 | 265 | 29.44 |
| 59 | Kaempferol and quercetin isolated from *Euonymus alatus* improve glucose uptake of 3T3-L1 cells without adipogenesis activity | Fang, Xian-Kang | Article | Life Sciences(5.3) | 2008 | 262 | 15.41 |
| 60 | Quercetin inhibits expression of inflammatory cytokines through attenuation of NF-κB and p38 MAPK in HMC-1 human mast cell line | Min, Y.-D. | Article | Inflammation Research(5.2) | 2007 | 262 | 14.56 |
| 61 | Effects of Low Dose Quercetin: Cancer Cell-Specific Inhibition of Cell Cycle Progression | Jeong, Jae-Hoon | Article | Journal of Cellular Biochemistry(3.1) | 2009 | 260 | 16.25 |
| 62 | Anti-inflammatory activity of quercetin and isoquercitrin in experimental murine allergic asthma | Rogerio, A. P. | Article | Inflammation Research(5.2) | 2007 | 260 | 14.44 |
| 63 | Absorption of quercetin-3-glucoside and quercetin-4′-glucoside in the rat small intestine: the role of lactase phlorizin hydrolase and the sodium-dependent glucose transporter | Day, AJ | Article | Biochemical Pharmacology(5.2) | 2003 | 260 | 11.82 |
| 64 | Quercetin derivatives are deconjugated and converted to hydroxyphenylacetic acids but not methylated by human fecal flora in vitro | Aura, AM | Article | Journal of Agricultural and Food Chemistry(6) | 2002 | 260 | 11.3 |
| 65 | Neuroprotective effects of antioxidative flavonoids, quercetin, (+)-dihydroquercetin and quercetin 3-methyl ether, isolated from *Opuntia ficus-indica var. saboten* | Dok-Go, H | Article | Brain Research(2.9) | 2003 | 257 | 11.68 |
| 66 | Simultaneous determination of catechin, rutin, quercetin kaempferol and isorhamnetin in the extract of sea buckthorn (*Hippophae rhamnoides L*.) leaves by RP-HPLC with DAD | Zu, Yuangang | Article | Journal of Pharmaceutical and Biomedical Analysis(3.1) | 2006 | 256 | 13.47 |
| 67 | Experimental determination of octanol-water partition coefficients of quercetin and related flavonoids | Rothwell, JA | Article | Journal of Agricultural and Food Chemistry(6) | 2005 | 254 | 12.7 |
| 68 | A novel voltammetric sensor based on gold nanoparticles involved in p-aminothiophenol functionalized multi-walled carbon nanotubes: Application to the simultaneous determination of quercetin and rutin | Yola, Mehmet Lutfi | Article | Electrochimica Acta(5.5) | 2014 | 253 | 23 |
| 69 | Intestinal transport of quercetin glycosides in rats involves both deglycosylation and interaction with the hexose transport pathway | Gee, JM | Article | Journal of Nutrition(4.3) | 2000 | 253 | 10.12 |
| 70 | Preparation, physicochemical and biological evaluation of quercetin based chitosan-gelatin film for food packaging | Yadav, Srasti | Article | Carbohydrate Polymers(10.2) | 2020 | 252 | 50.4 |
| 71 | Bacteriostatic Effect of Quercetin as an Antibiotic Alternative In Vivo and Its Antibacterial Mechanism In Vitro | Wang, Shengnan | Article | Journal of Food Protection(2.3) | 2018 | 251 | 35.86 |
| 72 | Quercetin downregulates NADPH oxidase, increases eNOS activity and prevents endothelial dysfunction in spontaneously hypertensive rats | Sánchez, M | Article | Journal of Hypertension(4.4) | 2006 | 250 | 13.16 |
| 73 | Role of quercetin as an alternative for obesity treatment: You are what you eat! | Nabavi, Seyed Fazel | Review | Food Chemistry(8.2) | 2015 | 242 | 24.2 |
| 74 | Quercetin-mediated Cell Cycle Arrest and Apoptosis Involving Activation of a Caspase Cascade through the Mitochondria! Pathway in Human Breast Cancer MCF-7 Cells | Chou, Chu-Chung | Article | Archives of Pharmacal Research(5.7) | 2010 | 239 | 15.93 |
| 75 | Quercetin decreases oxidative stress, NF-κB activation, and iNOS overexpression in liver of streptozotocin-induced diabetic rats | Dias, Alexandre Simoes | Article | Journal of Nutrition(4.3) | 2005 | 239 | 11.95 |
| 76 | Identification of quercetin 3-*O*-β-D-glucuronide as an antioxidative metabolite in rat plasma after oral administration of quercetin | Moon, JH | Article | Free Radical Biology and Medicine(7.9) | 2001 | 239 | 9.96 |
| 77 | Protective effect of topical formulations containing quercetin against UVB-induced oxidative stress in hairless mice | Casagrande, Rubia | Article | Journal of Photochemistry and Photobiology B-Biology(5.3) | 2006 | 237 | 12.47 |
| 78 | Quercetin hinders microglial activation to alleviate neurotoxicity via the interplay between NLRP3 inflammasome and mitophagy | Han, Xiaojuan | Article | Redox Biology(11.7) | 2021 | 230 | 57.5 |
| 79 | *In vivo* protective effects of quercetin against sodium fluoride-induced oxidative stress in the hepatic tissue | Nabavi, Seyed Mohammad | Article | Food Chemistry(8.2) | 2012 | 228 | 17.54 |
| 80 | Urinary and plasma levels of resveratrol and quercetin in humans, mice, and rats after ingestion of pure compounds and grape juice | Meng, XF | Article | Journal of Agricultural and Food Chemistry(6) | 2004 | 228 | 10.86 |
| 81 | Kinetics of mushroom tyrosinase inhibition by quercetin | Chen, QX | Article | Journal of Agricultural and Food Chemistry(6) | 2002 | 228 | 9.91 |
| 82 | Bioavailability and metabolism of the flavonol quercetin in the pig | Ader, P | Article | Free Radical Biology and Medicine(7.9) | 2000 | 227 | 9.08 |
| 83 | Quercetin, a flavonoid antioxidant, prevents and protects against ethanol-induced oxidative stress in mouse liver | Molina, MF | Article | Biological & Pharmaceutical Bulletin(1.9) | 2003 | 224 | 10.18 |
| 84 | Antioxidative and prooxidative effects of quercetin on A549 cells | Robaszkiewicz, Agnieszka | Article | Cell Biology International(3.6) | 2007 | 223 | 12.39 |
| 85 | Antihypertensive effects of the flavonoid quercetin | Perez-Vizcaino, Francisco | Review | Pharmacological Reports(3.2) | 2009 | 222 | 13.88 |
| 86 | Anti-biofilm activities of quercetin and tannic acid against *Staphylococcus aureus* | Lee, Jin-Hyung | Article | Biofouling(3.7) | 2013 | 221 | 18.42 |
| 87 | Quercetin content in some food and herbal samples | Wach, Anna | Article | Food Chemistry(8.2) | 2007 | 221 | 12.28 |
| 88 | Inhibition of iNOS gene expression by quercetin is mediated by the inhibition of IκB kinase, nuclear factor-kappa B and STAT1, and depends on heme oxygenase-1 induction in mouse BV-2 microglia | Chen, JC | Article | European Journal of Pharmacology(4.3) | 2005 | 219 | 10.95 |
| 89 | Inhibition of nitric oxide synthase inhibitors and lipopolysaccharide induced inducible NOS and cyclooxygenase-2 gene expressions by rutin, quercetin, and quercetin pentaacetate in RAW 264.7 macrophages | Chen, YC | Article | Journal of Cellular Biochemistry(3.1) | 2001 | 218 | 9.08 |
| 90 | Quercetin Remodels the Tumor Microenvironment To Improve the Permeation, Retention, and Antitumor Effects of Nanoparticles | Hu, Kaili. | Article | Acs Nano(16.2) | 2017 | 217 | 27.13 |
| 91 | Analysis of ellagitannins and conjugates of ellagic acid and quercetin in raspberry fruits by LC-MS^n^ | Mullen, W | Article | Phytochemistry(3.4) | 2003 | 216 | 9.82 |
| 92 | Determination of rutin and quercetin in plants by capillary electrophoresis with electrochemical detection | Chen, G | Article | Analytica Chimica Acta(5.5) | 2000 | 216 | 8.64 |
| 93 | Accumulation of orally administered quercetin in brain tissue and its antioxidative effects in rats | Ishisaka, Akari | Article | Free Radical Biology and Medicine(7.9) | 2011 | 215 | 15.36 |
| 94 | Bioavailabilities of quercetin-3-glucoside and quercetin-4′-glucoside do not differ in humans | Olthof, MR | Article; Proceedings Paper | Journal of Nutrition(4.3) | 2000 | 214 | 8.56 |
| 95 | Spectroscopic and structural study of complexes of quercetin with Al(III) | Cornard, JP | Article | Journal of Inorganic Biochemistry(3.5) | 2002 | 211 | 9.17 |
| 96 | Fabrication of stable zein nanoparticles coated with soluble soybean polysaccharide for encapsulation of quercetin | Li, Hao | Article | Food Hydrocolloids(11.3) | 2019 | 210 | 35 |
| 97 | Physicochemical characterization and antioxidant activity of quercetin-loaded chitosan nanoparticles | Zhang, Yuying | Article | Journal of Applied Polymer Science(2.8) | 2008 | 209 | 12.29 |
| 98 | Synthesis, characterization, antioxidative and antitumor activities of solid quercetin rare earth(III) complexes | Zhou, J | Article | Journal of Inorganic Biochemistry(3.5) | 2001 | 209 | 8.71 |
| 99 | Quercetin Inhibits Advanced Glycation End Product Formation by Trapping Methylglyoxal and Glyoxal | Li, Xiaoming | Article | Journal of Agricultural and Food Chemistry(6) | 2014 | 208 | 18.91 |
| 100 | The flavonoids quercetin and catechin synergistically inhibit platelet function by antagonizing the intracellular production of hydrogen peroxide | Pignatelli, P | Article | American Journal of Clinical Nutrition(7) | 2000 | 208 | 8.32 |
| 101 | A review on anti-cancer properties of Quercetin in breast cancer | Ezzati, Maryam | Review | Life Sciences(5.3) | 2020 | 207 | 41.4 |
| 102 | Effect of quercetin and its metabolites isorhamnetin and quercetin-3-glucuronide on inflammatory gene expression: role of miR-155 | Boesch-Saadatmandi, Christine | Article | Journal of Nutritional Biochemistry(5.4) | 2011 | 207 | 14.79 |
| 103 | Quercetin molecularly imprinted polymers: Preparation, recognition characteristics and properties as sorbent for solid-phase extraction | Song, Xingliang | Article | Talanta(5) | 2009 | 206 | 12.88 |
| 104 | The berry constituents quercetin, kaempferol, and pterostilbene synergistically attenuate reactive oxygen species: Involvement of the Nrf2-ARE signaling pathway | Saw, Constance Lay Lay | Article | Food and Chemical Toxicology(4.5) | 2014 | 205 | 18.64 |
| 105 | Molecular Targets Underlying the Anticancer Effects of Quercetin: An Update | Khan, Fazlullah | Review | Nutrients(5.8) | 2016 | 203 | 22.56 |
| 106 | Quercetin pharmacokinetics in humans | Moon, Young J. | Article | Biopharmaceutics & Drug Disposition(1.6) | 2008 | 203 | 11.94 |
| 107 | Co-encapsulation of Tamoxifen and Quercetin in Polymeric Nanoparticles: Implications on Oral Bioavailability, Antitumor Efficacy, and Drug-Induced Toxicity | Jain, Amit K. | Article | Molecular Pharmaceutics(4.6) | 2013 | 202 | 16.83 |
| 108 | Antibacterial and antioxidant activities of quercetin oxidation products from yellow onion (*Allium cepa*) skin | Ramos, Freddy A. | Article | Journal of Agricultural and Food Chemistry(6) | 2006 | 200 | 10.53 |
| 109 | The interaction of quercetin with human serum albumin: a fluorescence spectroscopic study | Sengupta, B | Article | Biochemical and Biophysical Research Communications(2.7) | 2002 | 198 | 8.61 |
| 110 | Quercetin-3-glucoside is transported by the glucose carrier SGLT1 across the brush border membrane of rat small intestine | Wolffram, S | Article | Journal of Nutrition(4.3) | 2002 | 198 | 8.61 |
| 111 | Molecular mechanisms underlying protective role of quercetin in attenuating Alzheimer's disease | Zaplatic, Elizabeta | Review | Life Sciences(5.3) | 2019 | 197 | 32.83 |
| 112 | Chronic dietary intake of quercetin alleviates hepatic fat accumulation associated with consumption of a Western-style diet in C57/BL6J mice | Kobori, Masuko | Article | Molecular Nutrition & Food Research(5.6) | 2011 | 196 | 14 |
| 113 | Quercetin-induced apoptosis acts through mitochondrial- and caspase-3-dependent pathways in human breast cancer MDA-MB-231 cells | Chien, Su-Yu | Article | Human & Experimental Toxicology(3) | 2009 | 196 | 12.25 |
| 114 | Evaluation of antiviral activities of *Houttuynia cordata Thunb*. extract, quercetin, quercetrin and cinanserin on murine coronavirus and dengue virus infection | Chiow, K. H. | Article | Asian Pacific Journal of Tropical Medicine(1.5) | 2016 | 193 | 21.44 |
| 115 | Quercetin Inhibit Human SW480 Colon Cancer Growth in Association with Inhibition of Cyclin D1 and Survivin Expression through Wnt/-Catenin Signaling Pathway | Shan, Bao-En | Article | Cancer Investigation(2.1) | 2009 | 193 | 12.06 |
| 116 | Neuroprotective effects of quercetin and rutin on spatial memory impairment in an 8-arm radial maze task and neuronal death induced by repeated cerebral ischemia in rats | Pu, Fengling | Article | Journal of Pharmacological Sciences(3) | 2007 | 192 | 10.67 |
| 117 | Direct HPLC analysis of quercetin and *trans*-resveratrol in red wine, grape, and winemaking byproducts | Careri, M | Article | Journal of Agricultural and Food Chemistry(6) | 2003 | 192 | 8.73 |
| 118 | Crystal structure of the copper-containing quercetin 2,3-dioxygenase from *Aspergillus japonicus* | Fusetti, F | Article | Structure(4.3) | 2002 | 192 | 8.35 |
| 119 | Quercetin and its derivates as antiviral potentials: A comprehensive review | Di Petrillo, Amalia | Review | Phytotherapy Research(7) | 2022 | 191 | 47.75 |
| 120 | Quercetin potentiates insulin secretion and protects INS-1 pancreatic β-cells against oxidative damage via the ERK1/2 pathway | Youl, E. | Article | British Journal of Pharmacology(7.4) | 2010 | 191 | 12.73 |
| 121 | Quercetin: A potential drug to reverse multidrug resistance | Chen, Chen | Review | Life Sciences(5.3) | 2010 | 191 | 12.73 |
| 122 | Quercetin and rutin exhibit antiamyloidogenic and fibril-disaggregating effects *in vitro* and potent antioxidant activity in APPswe cells | Jimenez-Aliaga, Karim | Article | Life Sciences(5.3) | 2011 | 190 | 13.57 |
| 123 | Protective effects of quercetin and vitamin C against oxidative stress-induced neurodegeneration | Heo, HJ | Article | Journal of Agricultural and Food Chemistry(6) | 2004 | 190 | 9.05 |
| 124 | Quercetin suppresses the mobility of breast cancer by suppressing glycolysis through Akt-mTOR pathway mediated autophagy induction | Jia, Lijun | Article | Life Sciences(5.3) | 2018 | 189 | 27 |
| 125 | Quercetin, but not rutin and quercitrin, prevention of H_2_O_2_-induced apoptosis via anti-oxidant activity and heme oxygenase 1 gene expression in macrophages | Chow, JM | Article | Biochemical Pharmacology(5.2) | 2005 | 189 | 9.45 |
| 126 | In vitro and in vivo inhibitory activities of rutin, wogonin, and quercetin on lipopolysaccharide-induced nitric oxide and prostaglandin E_2_ production | Shen, SC | Article | European Journal of Pharmacology(4.3) | 2002 | 189 | 8.22 |
| 127 | Quercetin attenuates oxidative stress-induced apoptosis via SIRT1/AMPK-mediated inhibition of ER stress in rat chondrocytes and prevents the progression of osteoarthritis in a rat model | Feng, Kai | Article | Journal of Cellular Physiology(5.3) | 2019 | 188 | 31.33 |
| 128 | Endogenous and exogenous mediators of quercetin bioavailability | Guo, Yi | Review | Journal of Nutritional Biochemistry(5.4) | 2015 | 187 | 18.7 |
| 129 | The type of sugar moiety is a major determinant of the small intestinal uptake and subsequent biliary excretion of dietary quercetin glycosides | Arts, ICW | Article | British Journal of Nutrition(3.5) | 2004 | 187 | 8.9 |
| 130 | Resveratrol and quercetin inhibit angiogenesis in vitro | Igura, K | Article | Cancer Letters(8.3) | 2001 | 187 | 7.79 |
| 131 | Quercetin and cancer: new insights into its therapeutic effects on ovarian cancer cells | Vafadar, Asma | Review | Cell and Bioscience(7) | 2020 | 186 | 37.2 |
| 132 | A minireview of quercetin: from its metabolism to possible mechanisms of its biological activities | Ulusoy, Hande Gul | Review | Critical Reviews in Food Science and Nutrition(10.3) | 2020 | 186 | 31 |
| 133 | Effects of Quercetin on Blood Pressure: A Systematic Review and Meta-Analysis of Randomized Controlled Trials | Serban, Maria-Corina | Review | Journal of the American Heart Association(5.7) | 2016 | 186 | 20.67 |
| 134 | Potential protective effects of quercetin and curcumin on paracetamol-induced histological changes, oxidative stress, impaired liver and kidney functions and haematotoxicity in rat | Yousef, Mokhtar I. | Article | Food and Chemical Toxicology(4.5) | 2010 | 186 | 12.4 |
| 135 | Solubility and solution thermodynamic properties of quercetin and quercetin dihydrate in subcritical water | Srinivas, Keerthi | Article | Journal of Food Engineering(5.3) | 2010 | 186 | 12.4 |
| 136 | Quercetin alleviates rat osteoarthritis by inhibiting inflammation and apoptosis of chondrocytes, modulating synovial macrophages polarization to M2 macrophages | Hu, Yue | Article | Free Radical Biology and Medicine(7.9) | 2019 | 185 | 30.83 |
| 137 | In vitro and ex vivo anti-inflammatory activity of quercetin in healthy volunteers | Boots, Agnes W. | Article | Nutrition(3.7) | 2008 | 185 | 10.88 |
| 138 | Quercetin Reduces High-Fat Diet-Induced Fat Accumulation in the Liver by Regulating Lipid Metabolism Genes | Jung, Chang Hwa | Article | Phytotherapy Research(7) | 2013 | 184 | 15.33 |
| 139 | The quercetin paradox | Boots, Agnes W. | Article | Toxicology and Applied Pharmacology(3.6) | 2007 | 183 | 10.17 |
| 140 | Quercetin ameliorates hyperglycemia and dyslipidemia and improves antioxidant status in type 2 diabetic db/db mice | Jeong, Soo-Mi | Article | Nutrition Research and Practice(2.1) | 2012 | 182 | 14 |
| 141 | Quercetin as a tyrosinase inhibitor: Inhibitory activity, conformational change and mechanism | Fan, Meihui | Article | Food Research International(7.4) | 2017 | 181 | 22.63 |
| 142 | DNA binding, cytotoxicity, apoptotic inducing activity, and molecular modeling study of quercetin zinc(II) complex | Tan, Jun | Article | Bioorganic & Medicinal Chemistry(3.1) | 2009 | 181 | 11.31 |
| 143 | Increase of stress resistance and lifespan of *Caenorhabditis elegans* by quercetin | Kampkoetter, Andreas | Article | Comparative Biochemistry and Physiology B-Biochemistry & Molecular Biology(2) | 2008 | 180 | 10.59 |
| 144 | Potent inhibitory effect of naturally occurring flavonoids quercetin and kaempferol on in vitro osteoclastic bone resorption | Wattel, A | Article | Biochemical Pharmacology(5.2) | 2003 | 180 | 8.18 |
| 145 | In vitro and in vivo evidence that quercetin protects against diabetes and its complications: A systematic review of the literature | Shi, Guang-Jiang | Review | Biomedicine & Pharmacotherapy(6.8) | 2019 | 179 | 29.83 |
| 146 | Quercetin induces apoptosis and autophagy in primary effusion lymphoma cells by inhibiting PI3K/AKT/mTOR and STAT3 signaling pathways | Granato, Marisa | Article | Journal of Nutritional Biochemistry(5.4) | 2017 | 179 | 22.38 |
| 147 | Development of a Quercetin-loaded nanostructured lipid carrier formulation for topical delivery | Guo Chen-yu | Article | International Journal of Pharmaceutics(5.6) | 2012 | 179 | 13.77 |
| 148 | A role for quercetin in coronavirus disease 2019 (COVID-19) | Derosa, Giuseppe | Review | Phytotherapy Research(7) | 2021 | 178 | 35.6 |
| 149 | Preparation and evaluation of quercetin-loaded lecithin-chitosan nanoparticles for topical delivery | Tan, Qi | Article | International Journal of Nanomedicine(7.5) | 2011 | 178 | 12.71 |
| 150 | Ingestion of quercetin inhibits platelet aggregation and essential components of the collagen-stimulated platelet activation pathway in humans | Hubbard, GP | Article | Journal of Thrombosis and Haemostasis(6.6) | 2004 | 178 | 8.48 |
| 151 | Advanced solid phase extraction using molecularly imprinted polymers for the determination of quercetin in red wine | Molinelli, A | Article | Journal of Agricultural and Food Chemistry(6) | 2002 | 178 | 7.74 |
| 152 | Fisetin and Quercetin: Promising Flavonoids with Chemopreventive Potential | Kashyap, Dharambir | Review | Biomolecules(5.4) | 2019 | 177 | 29.5 |
| 153 | Antioxidant activity of protein-bound quercetin | Rohn, S | Article | Journal of Agricultural and Food Chemistry(6) | 2004 | 177 | 8.43 |
| 154 | Quercetin-nanostructured lipid carriers: Characteristics and anti-breast cancer activities *in vitro* | Sun, Ming | Article | Colloids and Surfaces B-Biointerfaces(5.1) | 2014 | 176 | 16 |
| 155 | Quercetin and rutin prevent scopolamine-induced memory impairment in zebrafish | Richetti, S. K. | Article | Behavioural Brain Research(2.8) | 2011 | 176 | 12.57 |
| 156 | Myricetin, quercetin and catechin-gallate inhibit glucose uptake in isolated rat adipocytes | Strobel, P | Article | Biochemical Journal(3.7) | 2005 | 176 | 8.8 |
| 157 | Accumulation of quercetin conjugates in blood plasma after the short-term ingestion of onion by women | Moon, JH | Article | American Journal of Physiology-Regulatory Integrative and Comparative Physiology(2.8) | 2000 | 176 | 7.04 |
| 158 | Quercetin Alleviates Ferroptosis of Pancreatic β Cells in Type 2 Diabetes | Li, Dan | Article | Nutrients(5.8) | 2020 | 175 | 35 |
| 159 | Quercetin loaded biopolymeric colloidal particles prepared by simultaneous precipitation of quercetin with hydrophobic protein in aqueous medium | Patel, Ashok R. | Article | Food Chemistry(8.2) | 2012 | 174 | 13.38 |
| 160 | Quercetin, an anti-oxidant bioflavonoid, attenuates diabetic nephropathy in rats | Anjaneyulu, M | Article | Clinical and Experimental Pharmacology and Physiology(2.4) | 2004 | 173 | 8.24 |
| 161 | Quercetin and the mitochondria: A mechanistic view | de Oliveira, Marcos Roberto | Review | Biotechnology Advances(14.6) | 2016 | 172 | 19.11 |
| 162 | Quercetin, but not its glycosides, is absorbed from the rat stomach | Crespy, V | Article | Journal of Agricultural and Food Chemistry(6) | 2002 | 172 | 7.48 |
| 163 | Neuroprotective Effect of Quercetin Against the Detrimental Effects of LPS in the Adult Mouse Brain | Khan, Amjad | Article | Frontiers in Pharmacology(5) | 2018 | 171 | 24.43 |
| 164 | Preparation of a chemically stable quercetin formulation using nanosuspension technology | Gao, Lei | Article | International Journal of Pharmaceutics(5.6) | 2011 | 170 | 12.14 |
| 165 | Antiviral activity of quercetin 7-rhamnoside against porcine epidemic diarrhea virus | Choi, Hwa-Jung | Article | Antiviral Research(5) | 2009 | 170 | 10.63 |
| 166 | Safety of quercetin for clinical application (Review) | Okamoto, T | Review | International Journal of Molecular Medicine(4.4) | 2005 | 169 | 8.45 |
| 167 | Physicochemical and structural characterization of quercetin-β-cyclodextrin complexes | Zheng, Y | Article | Journal of Pharmaceutical Sciences(3.5) | 2005 | 169 | 8.45 |
| 168 | Silica/quercetin sol-gel hybrids as antioxidant dental implant materials | Catauro, Michelina | Article | Science and Technology of Advanced Materials(7.8) | 2015 | 167 | 16.7 |
| 169 | Effects of Quercetin on the Bioavailability of Doxorubicin in Rats: Role of CYP3A4 and P-gp Inhibition by Quercetin | Choi, Jun-Shik | Article | Archives of Pharmacal Research(5.7) | 2011 | 166 | 11.86 |
| 170 | Quercetin regulates Th1/Th2 balance in a murine model of asthma | Park, Hee-ju | Article | International Immunopharmacology(5) | 2009 | 166 | 10.38 |
| 171 | Quercetin and metabolic syndrome: A review | Hosseini, Azar | Review | Phytotherapy Research(7) | 2021 | 165 | 41.25 |
| 172 | Bioactive effects of quercetin in the central nervous system: Focusing on the mechanisms of actions | Suganthy, Natarajan | Review | Biomedicine & Pharmacotherapy(6.8) | 2016 | 165 | 18.33 |
| 173 | After cellular internalization, quercetin causes Nrf2 nuclear translocation, increases glutathione levels, and prevents neuronal death against an oxidative insult | Arredondo, Florencia | Article | Free Radical Biology and Medicine(7.9) | 2010 | 165 | 11 |
| 174 | Antiviral Activity of Baicalein and Quercetin against the Japanese Encephalitis Virus | Johari, Jefree | Article | International Journal of Molecular Sciences(5.6) | 2012 | 164 | 12.62 |
| 175 | Pharmacokinetics and modeling of quercetin and metabolites | Chen, X | Article | Pharmaceutical Research(3.8) | 2005 | 164 | 8.2 |
| 176 | Blockade of the epidermal growth factor receptor tyrosine kinase activity by quercetin and luteolin leads to growth inhibition and apoptosis of pancreatic tumor cells | Lee, LT | Article | Anticancer Research(1.8) | 2002 | 164 | 7.13 |
| 177 | Antimicrobial Activity of Quercetin: An Approach to Its Mechanistic Principle | Nguyen, Thi Lan Anh | Review | Molecules(4.6) | 2022 | 163 | 54.33 |
| 178 | Bioavailability of Quercetin in Humans with a Focus on Interindividual Variation | Filipa Almeida, A. | Review | Comprehensive Reviews in Food Science and Food Safety(15.9) | 2018 | 163 | 23.29 |
| 179 | Research Progress in the Modification of Quercetin Leading to Anticancer Agents | Massi, Alessandro | Review | Molecules(4.6) | 2017 | 162 | 20.25 |
| 180 | Hormesis and synergy: pathways and mechanisms of quercetin in cancer prevention and management | Vargas, Ashley J. | Review | Nutrition reviews(6.6) | 2010 | 162 | 10.8 |
| 181 | Anxiety and cognitive effects of quercetin liposomes in rats | Priprem, Aroonsri | Article | Nanomedicine-Nanotechnology Biology and Medicine(5) | 2008 | 162 | 9.53 |
| 182 | Quercetin, a dietary-derived flavonoid, possesses antiangiogenic potential | Tan, WF | Article | European Journal of Pharmacology(4.3) | 2003 | 162 | 7.36 |
| 183 | Enhancing oral bioavailability of quercetin using novel soluplus polymeric micelles | Dian, Linghui | Article | Nanoscale Research Letters(5.5) | 2014 | 160 | 14.55 |
| 184 | Anti-ageing and rejuvenating effects of quercetin | Chondrogianni, Niki | Article | Experimental Gerontology(3.8) | 2010 | 160 | 10.67 |
| 185 | Therapeutic and preventive properties of quercetin in experimental arthritis correlate with decreased macrophage inflammatory mediators | Mamani-Matsuda, Maria | Article | Biochemical Pharmacology(5.2) | 2006 | 159 | 8.37 |
| 186 | Quercetin reverses D-galactose induced neurotoxicity in mouse brain | Lu, Jun | Article | Behavioural Brain Research(2.8) | 2006 | 159 | 8.37 |
| 187 | Mulberry (*Morus alba L*.) leaves and their major flavonol quercetin 3-(6-malonylglucoside) attenuate atherosclerotic lesion development in LDL receptor-deficient mice | Enkhmaa, B | Article | Journal of Nutrition(4.3) | 2005 | 159 | 7.95 |
| 188 | Effects of the pure flavonoids epicatechin and quercetin on vascular function and cardiometabolic health: a randomized, double-blind, placebo-controlled, crossover trial | Dower, James I. | Article | American Journal of Clinical Nutrition(7) | 2015 | 158 | 15.8 |
| 189 | Anti-inflammatory effect of quercetin-loaded microemulsion in the airways allergic inflammatory model in mice | Rogerio, Alexandre P. | Article | Pharmacological Research(9) | 2010 | 158 | 10.53 |
| 190 | Quercetin 3-O-β-glucoside is better absorbed than other quercetin forms and is not present in rat plasma | Morand, C | Article | Free Radical Research(4.3) | 2000 | 158 | 6.32 |
| 191 | Complexation of quercetin with three kinds of cyclodextrins: An antioxidant study | Jullian, Carolina | Article | Spectrochimica Acta Part A-Molecular and Biomolecular Spectroscopy(3.8) | 2007 | 157 | 8.72 |
| 192 | Effects of *Biota orientalis* extract and its flavonoid constituents, quercetin and rutin on serum uric acid levels in oxonate-induced mice and xanthine dehydrogenase and xanthine oxidase activities in mouse liver | Zhu, JX | Article | Journal of Ethnopharmacology(4.8) | 2004 | 157 | 7.48 |
| 193 | Heat-induced, metal-catalyzed oxidative degradation of quercetin and rutin (quercetin 3-*O*-rhamnosylglucoside) in aqueous model systems | Makris, DP | Article | Journal of Agricultural and Food Chemistry(6) | 2000 | 157 | 6.28 |
| 194 | Quercetin: a natural compound for ovarian cancer treatment | Shafabakhsh, Rana | Review | Journal of Ovarian Research(4.2) | 2019 | 156 | 26 |
| 195 | Protective role of quercetin against lead-induced inflammatory response in rat kidney through the ROS-mediated MAPKs and NF-κB pathway | Liu, Chan-Min | Article | Biochimica et Biophysica Acta-General Subjects(3) | 2012 | 156 | 12 |
| 196 | Quercetin inhibits human breast cancer cell proliferation and induces apoptosis via Bcl-2 and Bax regulation | Duo, Jian | Article | Molecular Medicine Reports(3) | 2012 | 156 | 12 |
| 197 | Quercetin downregulates matrix metalloproteinases 2 and 9 proteins expression in prostate cancer cells (PC-3) | Vijayababu, M. R. | Article | Molecular and Cellular Biochemistry(3.3) | 2006 | 156 | 8.21 |
| 198 | Quercetin activates AMP-activated protein kinase by reducing PP2C expression protecting old mouse brain against high cholesterol-induced neurotoxicity | Lu, Jun | Article | Journal of Pathology(7.1) | 2010 | 155 | 10.33 |
| 199 | Flavonoid quercetin differentiation decreases osteoclastic induced by RANKL via a mechanism involving NFκB and AP-1 | Wattel, A | Article | Journal of Cellular Biochemistry(3.1) | 2004 | 155 | 7.38 |
| 200 | Quercetin and allopurinol reduce liver thioredoxin-interacting protein to alleviate inflammation and lipid accumulation in diabetic rats | Wang, Wei | Article | British Journal of Pharmacology(7.4) | 2013 | 154 | 12.83 |

NO.(Rank according to WoS citation count); Type(Document Type); IF(Journal Impact factor Five Year); Year(Publication Year); Citations(Times Cited, WoS Core); ACY*(Average citations per year)

| **TABLE S2** Authors of the 200 most-cited articles on Quercetin (number of articles ≥ 3). | | | | | |
| --- | --- | --- | --- | --- | --- |
| **NO.** | **Author** | **Number of articles** | **Citation** | **Institutions** | **Country** |
| 1 | Wolffram, Siegfried | 6 | 1643 | University of Kiel | Germany |
| 2 | Nabavi, Seyed Mohammad | 5 | 1010 | Baqiyatallah University of Medical Sciences | Iran |
| 3 | Terao, Junji | 5 | 1600 | Tokushima University | Japan |
| 4 | Williamson, Gary | 5 | 936 | University of Leeds | England |
| 5 | Hollman, PCH | 4 | 1192 | Wageningen University & Research | Netherlands |
| 6 | Nabavi, Seyed Fazel | 4 | 807 | Baqiyatallah University of Medical Sciences | Iran |
| 7 | Bast, Aalt | 3 | 1777 | Maastricht University | Netherlands |
| 8 | Boots, Agnes W. | 3 | 1777 | Maastricht University | Netherlands |
| 9 | Haenen, Guido R. M. M. | 3 | 1777 | Maastricht University | Netherlands |
| 10 | Gonzalez-Gallego, Javier | 3 | 1008 | Universidad de Leon | Spain |
| 11 | Zhai Guang-xi | 3 | 877 | Shandong University | China |
| 12 | Duarte, Juan | 3 | 828 | University of Granada | Spain |
| 13 | Chen, YC | 3 | 596 | Taipei Medical University | Taiwan |
| 14 | Shen, SC | 3 | 596 | Taipei Medical University | Taiwan |
| 15 | Day, AJ | 3 | 767 | University of Leeds | England |

| **TABLE S3** The basic information of the major journals. | | | | | |
| --- | --- | --- | --- | --- | --- |
| **NO.** | **Journal** | **Publishers** | **IF(2023)** | **IF(5 Years)** | **JCR** |
| 1 | Food chemistry | Elsevier Science Incs | 8.5 | 8.2 | Q1 |
| 2 | Free radical biology and medicine | Elsevier Science Incs | 7.1 | 7.9 | Q1 |
| 3 | Phytotherapy research | Wiley | 6.1 | 7 | Q1 |
| 4 | Journal of agricultural and food chemistry | American Chemical Society | 5.7 | 6 | Q1 |
| 5 | Nutrients | Multidisciplinary Digital Publishing Institute | 4.8 | 5.8 | Q1 |
| 6 | Journal of nutritional biochemistry | Elsevier Science Incs | 4.8 | 5.4 | Q1 |
| 7 | Journal of nutrition | Elsevier Science Incs | 4.7 | 4.3 | Q1 |
| 8 | Life sciences | Pergamon-Elsevier Science Itd | 5.2 | 5.3 | Q1 |
| 9 | Biochemical pharmacology | Pergamon-Elsevier Science Itd | 5.3 | 5.2 | Q1 |
| 10 | Molecules | Multidisciplinary Digital Publishing Institute | 4.2 | 4.6 | Q2 |
| 11 | European journal of pharmacology | Elsevier | 4.2 | 4.3 | Q1 |
| 12 | British journal of nutrition | Cambridge University Press | 3 | 3.5 | Q2 |

| **TABLE S4** Classification of research topics in the 200 most-cited quercetin articles. | |
| --- | --- |
| **NO.** | **Articles topics (n = Number of articles ; citations) / Articles NO. (Rank according to WoS citation count)** |
| 1 | Cancer related (n = 31; 7271) **/** 8 ; 17 ; 27 ; 32 ; 40 ; 42 ; 43 ; 52 ; 61 ; 74 ; 90 ; 101 ; 105 ; 107 ; 113 ; 115 ; 121 ; 124 ; 130 ; 131 ; 146 ; 152 ; 154 ; 169 ; 176 ; 179 ; 180 ; 182 ; 194 ; 196 ; 197 |
| 2 | Anti-oxidant related (n = 20; 6513) **/** 1 ; 4 ; 10 ; 25 ; 26 ; 53 ; 77 ; 79 ; 83 ; 84 ; 93 ; 104 ; 108 ; 125 ; 134 ; 139 ; 153 ; 161 ; 169 ; 191 |
| 3 | Neurological disease related (n = 17; 3544) **/** 39 ; 46 ; 54 ; 65 ; 78 ; 88 ; 111 ; 116 ; 122 ; 123 ; 155 ; 163 ; 172 ; 173 ; 179 ; 186 ; 198 |
| 4 | Inflammation and Immunity related (n = 13; 4152) **/** 2 ; 11 ; 22 ; 55 ; 56 ; 60 ; 62 ; 89 ; 126 ; 137 ; 170 ; 185 ; 189 |
| 5 | Pharmacokinetics related (n = 12; 3334) **/** 19 ; 20 ; 21 ; 30 ; 44 ; 67 ; 80 ; 82 ; 106 ; 162 ; 175 ; 178 |
| 6 | Quercetin derivatives related (n = 12; 2483) **/** 63 ; 64 ; 69 ; 76 ; 94 ; 102 ; 110 ; 129 ; 150 ; 165 ; 187 ; 190 |
| 7 | Diabetes related (n = 11; 2801) **/** 7 ; 16 ; 59 ; 75 ; 99 ; 120 ; 140 ; 145 ; 158 ; 160 ; 200 |
| 8 | Nanotechnology related (n = 10; 2745) **/** 13 ; 24 ; 35 ; 51 ; 68 ; 96 ; 97 ; 147 ; 149 ; 164 |
| 9 | Quantitative analysis related (n = 9; 2547) **/** 3 ; 66 ; 87 ; 91 ; 92 ; 103 ; 117 ; 151 ; 193 |
| 10 | Cardiovascular disease related (n = 9; 2498) **/** 15 ; 33 ; 34 ; 48 ; 72 ; 85 ; 100 ; 128 ; 133 |
| 11 | Quercetin complexes related (n = 9; 1871) **/** 49 ; 70 ; 95 ; 98 ; 118 ; 135 ; 142 ; 159 ; 167 |
| 12 | Anti-viral related (n = 6; 1321) **/** 38 ; 58 ; 114 ; 119 ; 148 ; 174 |
| 13 | Therapeutic applications review related (n = 5; 2341) **/** 6 ; 9 ; 12 ; 29 ; 132 |
| 14 | Improved bioavailability related (n = 4; 1282) **/** 14 ; 41 ; 50 ; 183 |
| 15 | Bone disease related (n = 4; 708) **/** 127 ; 136 ; 144 ; 199 |
| 16 | Anti-ageing related (n = 3; 1040) **/** 5 ; 143 ; 184 |
| 17 | Dietary Supplement related (n = 3; 890) **/** 28 ; 36 ; 157 |
| 18 | Anti-obesity related (n = 3; 829) **/** 23 ; 73 ; 138 |
| 19 | Clinical application/research (n = 3; 748) **/** 18 ; 166 ; 188 |
| 20 | Anti-bacterial related (n = 3; 635) **/** 71 ; 86 ; 177 |
| 21 | Liver disease related (n = 2; 674) **/** 31 ; 47 |
| 22 | Interaction with human serum albumin related (n = 2; 517) **/** 45 ; 109 |
| 23 | Renal disease related (n = 2; 487) **/** 37 ; 195 |
| 24 | Quercetin as a tyrosinase inhibitor related (n = 2; 409) **/** 81 ; 141 |
| 25 | Metabolic syndrome related (n = 2; 361) **/** 112 ; 171 |
| 26 | Neuroprotective and Anticancer effects (n = 1; 267) / 57 |
| 27 | Hyperuricemia related (n = 1; 157) **/** 192 |
| 28 | Other (n=1; 176) **/** 156 |

| **TABLE S5** Classification of research areas in the 200 most-cited quercetin articles. | |
| --- | --- |
| **NO.** | **Research Areas (n = Number of articles ; citations) / Articles NO. (Rank according to WoS citation count)** |
| 1 | Biochemistry & Molecular Biology (n = 40 ; 9997) **/** 4 , 16 , 21 , 23 , 26 , 27 , 31 , 39 , 50 , 52 , 54 , 55 , 61 , 76 , 77 , 78 , 82 , 89 , 91 , 93 , 95 , 98 , 102 , 109 , 118 , 128 , 131 , 136 , 142 , 143 , 146 , 152 , 156 , 173 , 174 , 177 , 179 , 190 , 195 , 199 |
| 2 | Pharmacology & Pharmacy (n = 39 ; 11208) **/** 1 , 7 , 9 , 11 , 12 , 17 , 20 , 22 , 33 , 34 , 45 , 51 , 63 , 74 , 83 , 85 , 88 , 106 , 116 , 119 , 120 , 125 , 126 , 138 , 139 , 144 , 147 , 148 , 160 , 163 , 164 , 165 , 167 , 169 , 171 , 182 , 185 , 189 , 200 |
| 3 | Nutrition & Dietetics (n = 20 ; 6268 ) **/** 2 , 6 , 15 , 18 , 19 , 28 , 44 , 69 , 75 , 94 , 100 , 105 , 110 , 129 , 137 , 140 , 158 , 180 , 187 , 188 |
| 4 | Agriculture, Multidisciplinary (n = 14 ;3738) **/** 3 , 25 , 64 , 67 , 80 , 81 , 99 , 108 , 117 , 123 , 151 , 153 , 162 , 193 |
| 5 | Chemistry (n = 13 ; 3203 ) **/** 13 , 49 , 66 , 70 , 73 , 79 , 87 , 90 , 92 , 96 , 103 , 159 , 175 |
| 6 | Research & Experimental Medicine (n = 12 ; 2631) **/** 32 , 41 , 59 , 101 , 107 , 111 , 121 , 122 , 124 , 145 , 166 , 172 |
| 7 | Food Science & Technology (n = 10 ; 2887) **/** 10 , 14 , 30 , 36 , 104 , 112 , 132 , 134 , 141 , 178 |
| 8 | Oncology ( n = 8 ; 2065 ) **/** 8 , 40 , 43 , 115 , 130 , 176 , 196 , 198 |
| 9 | Cell Biology (n = 7 ; 1683) **/** 42 , 56 , 60 , 62 , 84 , 127 , 197 |
| 10 | Science & Technology - Other Topics (n = 4 ; 831) **/** 37 , 149 , 181 , 183 |
| 11 | Cardiovascular System & Cardiology (n = 3 ; 736) **/** 48 , 72 , 133 |
| 12 | Biotechnology & Applied Microbiology (n = 3 ; 644) **/** 71 , 86 , 161 |
| 13 | Electrochemistry (n=2 ; 654 ) **/** 24 , 68 |
| 14 | Neurosciences & Neurology (n = 2; 572) **/** 46 , 65 |
| 15 | Immunology (n=2; 496) **/** 38 , 170 |
| 16 | Behavioral Sciences (n = 2; 335) **/** 155 , 186 |
| 17 | Biophysics (n = 2 ; 516) **/** 35 , 154 |
| 18 | Spectroscopy (n = 2 ; 438) **/** 53 , 191 |
| 19 | Plant Sciences (n = 2 ; 424) **/** 57 , 192 |
| 20 | General & Internal Medicine (n = 1 ;700) **/** 5 |
| 21 | Endocrinology & Metabolism (n = 1 376 ) / 29 |
| 22 | Gastroenterology & Hepatology (n = 1 ; 314) / 47 |
| 23 | Virology ( n = 1 ; 265) / 58 |
| 24 | Polymer Science (n = 1 ; 209) / 97 |
| 25 | Toxicology (n = 1 ; 196) / 113 |
| 26 | Public, Environmental & Occupational Health (n = 1 ; 194) **/** 114 |
| 27 | Engineering (n=1 ; 186) / 135 |
| 28 | Geriatrics & Gerontology (n=1 ;184) / 184 |
| 29 | Hematology; Cardiovascular System & Cardiology (n=1 ; 178) / 150 |
| 30 | Physiology (n = 1 ; 176) / 157 |
| 31 | Materials Science (n=1;167) / 168 |
| 32 | Reproductive Biology (n = 1 ; 156) / 194 |

**FIGURE S1**
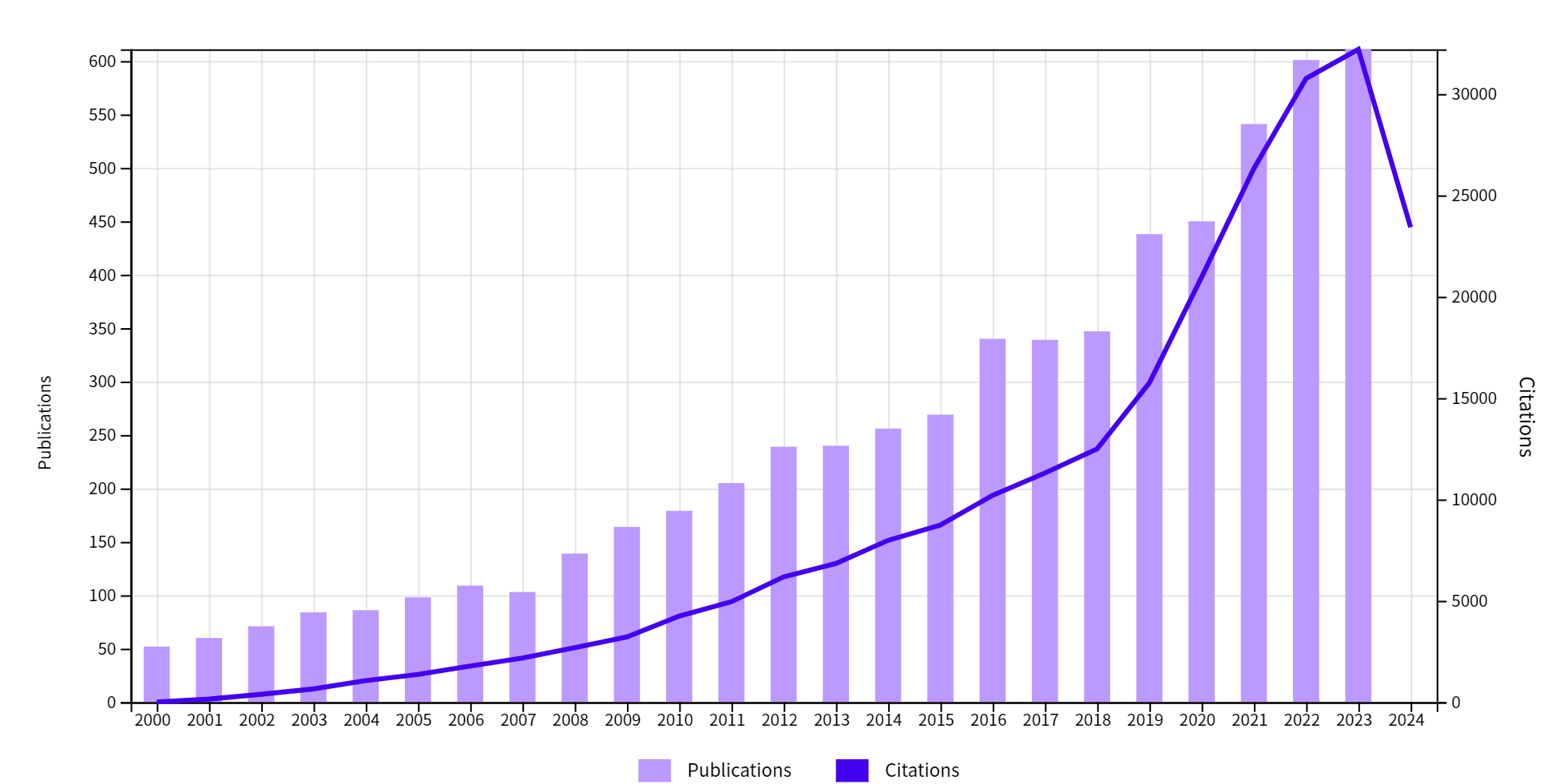


**FIGURE S2
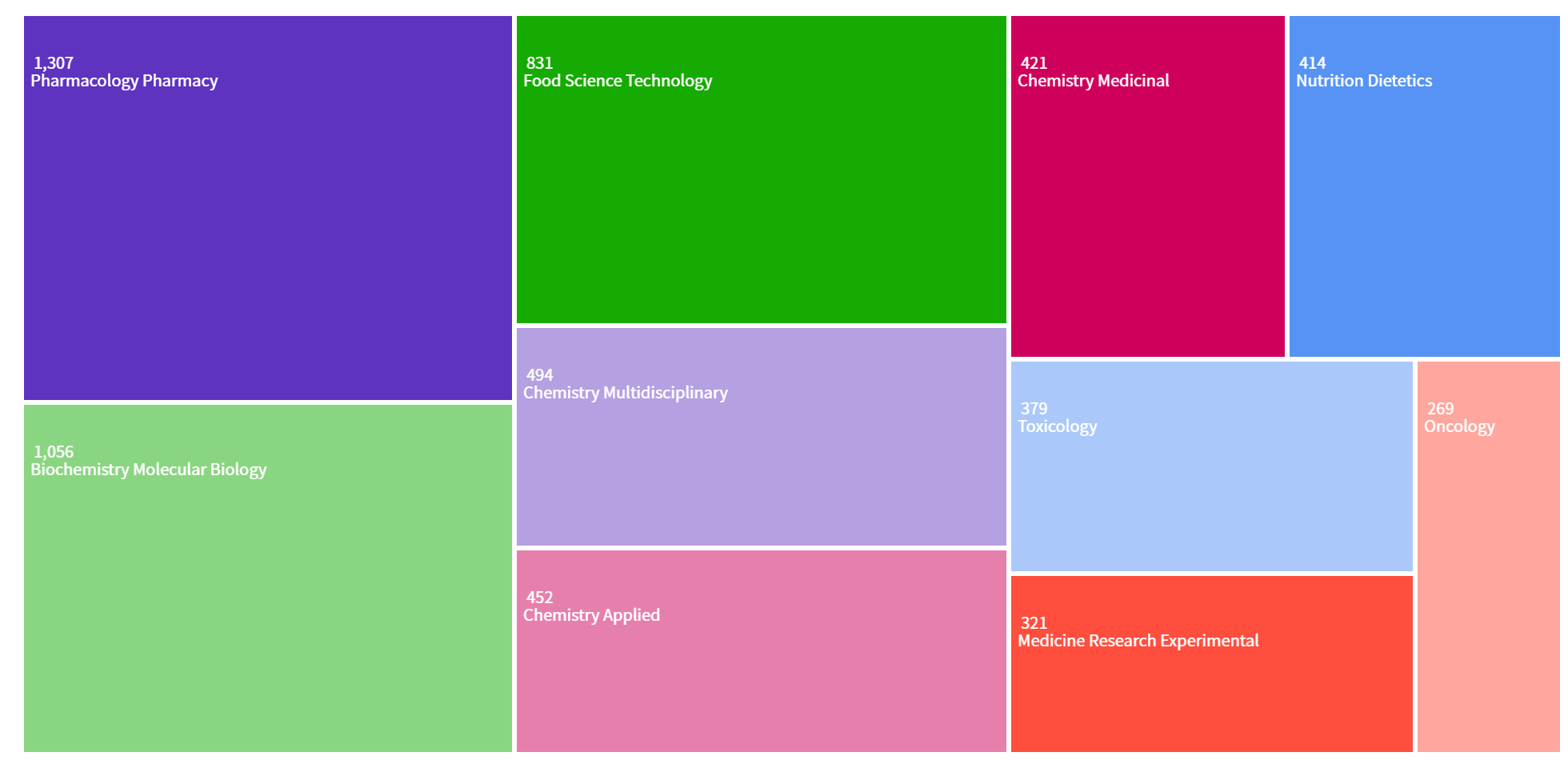
**
